# Biogenic Silver–Selenium nanocomposite with anticancer activity and potent efficacy against vancomycin-resistant *Staphylococcus aureus*

**DOI:** 10.1371/journal.pone.0351844

**Published:** 2026-07-02

**Authors:** Abeer S. Aloufi, Mohammed S. Abdulrahman, Amr H. Hashem, Ebrahim Saied, Ahmed Abdel Tawab, Faisal Alsenani, Adnan Alharbi, Sami I. Alzarea, Fathy M. Elkady

**Affiliations:** 1 Department of Biology, College of Science, Princess Nourah bint Abdulrahman University, Riyadh, Saudi Arabia; 2 Microbiology and Immunology Department, Faculty of Pharmacy (Boys), Al-Azhar University, Cairo, Egypt; 3 Department of Microbiology and Immunology, Faculty of Pharmacy, Menoufia National University, Menoufia, Egypt; 4 Botany and Microbiology Department, Faculty of Science, Al-Azhar University, Cairo, Egypt; 5 Department of Microbiology and Immunology, Faculty of Medicine, Al-Azhar University, Cairo, Egypt; 6 Department of Pharmaceutical Sciences, College of Pharmacy, Umm Al-Qura University, Makkah, Saudi Arabia; 7 Pharmaceutical Practices Department, College of Pharmacy, Umm Al-Qura University, Makkah, Saudi Arabia; 8 Department of Pharmacology, College of Pharmacy, Jouf University, Sakaka, Aljouf, Saudi Arabia; Mekdela Amba University, ETHIOPIA

## Abstract

The rapid emergence of multi-drug resistant (MDR) pathogenic bacteria as well as the continued burden of malignant diseases requires the safe development of novel and multifunctional therapeutic agents. The WHO designated vancomycin-resistant *Staphylococcus aureus* (VRSA) as “high priority” AMR pathogen. Thus, this study aimed to ecofriendly synthesis of silver–selenium nanocomposite (Ag–Se NC) and evaluate its *in vitro* anticancer effect and inhibitory activity against VRSA clinical isolates. Biogenic Ag–Se NC was successfully synthesized using the aqueous peel extract of *Cucumis melo* (*C. melo*) through an eco-friendly green synthesis approach. Following visual color transformation of the preparation mixture, nanocomposite formation was validated based on comprehensive physicochemical characterization using different spectroscopic analyses. The greenly synthesized Ag–Se NC revealed the Ag and Se specific surface plasmon resonance (SPR) peaks, high crystallinity, and predominantly spherical morphology with an average particle size of ~35 nm. Biological evaluations revealed that Ag–Se NC possesses selective cytotoxicity, displaying low toxicity toward WI-38 normal lung fibroblasts (IC₅₀ = 203.4 µg/mL) while exerting a potent, concentration-reliant inhibitory effect towards malignant cell lines, including hepatocellular carcinoma (Hep-G2) and breast adenocarcinoma (MCF-7) with IC50 90.97 and 38.18 μg/mL respectively. Furthermore, the Ag–Se NC demonstrated appreciated antibacterial activity, in comparison with the linezolid standard antimicrobial agent, against 11 VRSA clinical isolates, with MIC values ranging from 64 to 512 µg/mL and a mean MIC of 203.64 µg/mL. The marked NC bactericidal effects were indicated by their minimum inhibitory concentration index (MICi) values of 1–4, rapid time-kill kinetics, and significant membrane disruption evidenced by the protein leakage assay. The obtained NC also exhibited a respected inhibitory effect against VRSA biofilm development, in the range of 34.68 ± 2.4–72.89 ± 1.87%, as well as a strain-dependent partial eradication effect on the fully formed bacterial biofilm, ranging from 16.81 ± 0.96 to 42.59 ± 0.78%. Notably, variable interactions were observed when Ag–Se NC was combined with vancomycin against VRSA isolates; one isolate showed synergistic interaction with fractional inhibitory concentration index (FICi) = 0.5 and three isolates exhibited additive effects (FICi ranged from 0.5 to 1). In conclusion, these findings highlight Ag–Se NC as a promising green-synthesized nanoplatform with combined anticancer and bacterial inhibitory effects in both planktonic and biofilm growth forms and antimicrobial–potentiating activities.

## 1 Introduction

Microbial resistance is estimated to cause an annual death of about 10 million by 2050 [1]. *Staphylococcus aureus* (*S. aureus*) is a significant etiological agent that is responsible for a variety of human infections associated with mild skin diseases, fatal invasive diseases like septicemia, pneumonia, and infective endocarditis, as well as diseases mediated by microbial toxins, including toxic shock syndrome and food poisoning [2–4]. Treatment of such infections is mostly hindered by antimicrobial resistance (AMR) resulting from the indiscriminate antimicrobial usage [5]. *S. aureus* is a significant etiological agent responsible for a wide spectrum of human infections, ranging from mild skin diseases to life-threatening invasive conditions such as septicemia and infective endocarditis. Vancomycin serves as the last-resort antibiotic and cornerstone therapy for controlling serious infections caused by MDR Gram-positive pathogens, particularly MRSA [6]. The emergence of VRSA strains represents a critical global public health threat, as it effectively eliminates the most reliable therapeutic option against MDR staphylococcal infections. Furthermore, the World Health Organization (WHO) has designated VRSA as a “high priority” AMR pathogen due to its significant public health impact. Furthermore, the lack of sufficient treatment options for VRSA is associated with significant worldwide morbidity and mortality, which underscores the urgent need for novel alternative therapeutic strategies [7]. Despite that, VRSA strains are a global public health threat because of their spread and risk. Accordingly, the lack of sufficient treatment options and inhibition for these strains is associated with significant worldwide morbidity and mortality [8]. The World Health Organization (WHO) has listed VRSA as a “high priority AMR pathogen” because of its significant public health impact [1].

The dissatisfaction with conventional antimicrobial agents gives rise to various unique nanotherapeutic alternatives [9]. These nanomaterials have promising antibacterial activity via the release of metal ions and production of hydroxyl and superoxide reactive oxygen species (ROS), which efficiently destroy microbial cells [10]. The improved physico-chemical nanomaterials’ capabilities were raised from their particles’ minute size, effective shape, and composition [11]. Accordingly, nanoparticles (NPs) could possess unique antimicrobial activities or be able to deliver and improve the effectiveness of traditionally used antimicrobials. Additionally, their surface functionalization facilitates polyvalent interactions with various microbial biomolecules [12].

The distinctive NPs characters are primarily associated with their elevated surface/volume ratio, which changes the catalytic, thermal, and mechanical behaviors in comparison with their bulky counterparts. Bimetallic NCs (BNCs) are generally formed via successive or co-reduction of the precursors of both target metals [13]. Amongst the tested noble metal NPs, Se and Ag are broadly researched for their various biomedical applications. Silver NPs are well-known for their microbial inhibitory effect, water purification, food packaging industry, textiles, contact lenses, cosmetics, wound dressings, and implants, while Se NPs are extensively explored for diagnostic and cancer therapy uses. Their strong absorption of visible light and capacity for precise energy delivery make Se NPs suitable for photodynamic therapy and bioimaging applications [14,15].

The nanoscale size of these particles facilitates efficient cellular entry, supporting their use in targeted therapies. These NPs can be prepared by physical, chemical, or biological methods, with chemical synthesis being most common [16].

Combining silver and selenium into a bimetallic nanocomposite is expected to provide synergistic therapeutic advantages over monometallic nanoparticles. Silver nanoparticles are well-known for their strong antimicrobial activity but are often associated with dose-dependent cytotoxicity toward normal mammalian cells. Selenium nanoparticles, on the other hand, exhibit lower toxicity and possess antioxidant and anticancer properties [17]. The incorporation of selenium into silver-based nanostructures can therefore reduce silver-associated cytotoxicity while maintaining or enhancing antimicrobial efficacy. Additionally, the bimetallic configuration may facilitate increased reactive oxygen species (ROS) generation and improved biofilm disruption compared to monometallic counterparts, offering a multifunctional platform with superior biological performance [18]. The biological performance of Ag–Se bimetallic nanocomposites is highly influenced by their physicochemical properties. Parameters such as particle size, morphology, and surface charge directly affect cellular uptake, antimicrobial efficacy, biofilm penetration, and stability [19]. Optimizing these characteristics is essential to maximize therapeutic activity while minimizing cytotoxicity toward normal cells.

Several strategies have been reported for the synthesis of Ag–Se nanostructures. Chemical methods, including oil–water interface synthesis, sol-gel, template-assisted deposition, and laser ablation, have produced Ag–Se nanoparticles with well-defined morphologies and optical or electrical properties [20–22]. Although these approaches are effective, they often require harsh reaction conditions or toxic reagents, limiting their biocompatibility. Biological or green methods using plant extracts or bacterial cellulose have emerged as safer alternatives, providing cost-effective and environmentally friendly routes for nanoparticle synthesis [23,24]. Previous studies have demonstrated the antibacterial potential of Ag–Se nanocomposites against model Gram-positive and Gram-negative bacteria; however, their activity against multidrug-resistant pathogens, particularly vancomycin-resistant Staphylococcus aureus (VRSA), has been insufficiently explored [23,24]. Moreover, most reports focused on single biological or physicochemical properties, lacking comprehensive evaluations that integrate antibacterial, antibiofilm, cytotoxicity, and synergistic effects with conventional antibiotics.

In addition, concerns regarding the cytotoxicity of monometallic silver nanoparticles remain unresolved, and strategies to mitigate such toxicity, such as the incorporation of selenium, have not been systematically addressed [25]. Finally, the utilization of agro-waste-derived plant extracts, which can serve as efficient reducing and stabilizing agents, remains limited, highlighting the novelty of the present work in employing *Cucumis melo* peel extract to produce biocompatible and multifunctional Ag–Se bimetallic nanocomposites.

Many biological systems, including bacteria, fungi, and plants, are continuously assessed for the biosynthesis of various metallic NPs [26]. These systems provide a cost-effective, eco-friendly, and biocompatible alternative to traditional synthetic techniques [27]. Compared with conventional chemical synthesis, green synthesis using plant extracts offers several expected advantages. First, the phytochemicals in the extracts act as natural reducing and stabilizing agents, which enhance the stability of the resulting nanocomposites [28]. Second, these biologically derived capping agents improve the biocompatibility of the nanoparticles, reducing cytotoxic effects on normal mammalian cells [29]. Finally, the synergistic interactions between the metal core and the bioactive phytochemicals can enhance the biological performance, including antimicrobial, antibiofilm, and antioxidant activities [30]. These advantages make green-synthesized nanocomposites more suitable for biomedical applications than their chemically synthesized counterparts.

However, biosynthesized NPs using plant-derived materials are mostly preferred due to their ready availability, safe handling, rapid biosynthesis, not required cell culture maintenance, appropriateness for large-scale production, and ecofriendly. Additionally, plant extracts have various phytochemical classes, which could serve as capping agents for NPs [31]. Also, production of NPs using plant extracts is the best preparation method due to the high biosafety of the plant-based biosynthesized NPs [32]. Additionally, biological approaches offer better control over NPs size, morphology, and safety [33]. The green manufacture based on the extracts from different plant parts has appeared as a sustainable and more safe option, where the NPs reduction and stabilization are carried out by the phytochemicals, which improve their compatibility and activity [34,35]. Plant-derived metabolites such as flavonoids, alkaloids, terpenoids, phenolics, and vitamins play essential roles in metal reduction and stabilization [36].

Among the various plant-mediated synthesis approaches, the selection of *C. melo* peel extract in this study represents a novel and strategic choice. Unlike commonly used plant extracts, *C. melo* peel is an agricultural waste material rich in bioactive phytochemicals such as phenolics, flavonoids, and vitamins, which can act as efficient reducing and stabilizing agents during nanoparticle synthesis [37]. This unique phytochemical profile is expected to enhance the stability, biocompatibility, and biological activity of the resulting nanocomposites. Despite these advantages, the use of *C. melo* peel extract for the synthesis of Ag–Se bimetallic nanocomposites has not been sufficiently explored, highlighting its novelty and potential in developing sustainable and high-performance nanotherapeutics.

Metallic nanocomposites offer several strategic advantages that make them effective adjuvants or alternatives for vancomycin. First, because nanocomposites generate reactive oxygen species (ROS), physically damage bacterial cell membranes, and obstruct DNA replication and protein synthesis, it is extremely difficult for bacteria to build resistance through single-point mutations [38]. Second, because of their nanoscale size and high surface area-to-volume ratio, nanoparticles can successfully infiltrate and disrupt biofilm architecture, in contrast to traditional antibiotics that frequently fail to penetrate the protective extracellular polymeric matrix of bacterial biofilms [39]. Third, the functionality of medications like vancomycin against resistant germs can be restored by functionalizing nanoparticles to act as carriers that improve the transport and boost the effectiveness of currently available antibiotics [40].

Several factors highlight the novelty of the present study. First, this work explores, for the first time, the green synthesis of Ag–Se bimetallic nanocomposites using *C. melo* peel extract as an agro-waste-derived reducing and stabilizing agent, offering a sustainable and cost-effective alternative to conventional plant-mediated synthesis approaches. The rich phytochemical composition of *C. melo* peel, including phenolics and flavonoids, is expected to enhance nanoparticle stability and biological performance [41]. Second, while previous studies have primarily focused on monometallic nanoparticles or limited biological evaluations, this study provides a comprehensive assessment of Ag–Se nanocomposites against vancomycin-resistant Staphylococcus aureus (VRSA), a WHO-designated high-priority pathogen that remains insufficiently explored in the context of bimetallic nanomaterials [42].

Third, the incorporation of selenium into silver-based nanostructures is addressed as a strategy to mitigate the well-documented cytotoxicity of Ag nanoparticles toward normal mammalian cells, thereby improving their safety profile [43]. Finally, this study integrates multiple biological assays, including antibacterial, antibiofilm, time-kill kinetics, membrane integrity, and antibiotic synergy evaluations, in addition to anticancer activity, providing a unified and multifunctional investigation that is rarely reported in a single study.

Despite the extensive investigation of silver and selenium nanoparticles as individual antimicrobial and anticancer agents, studies addressing the biological performance of Ag–Se bimetallic nanocomposites remain limited, particularly against high-priority pathogens such as vancomycin-resistant *Staphylococcus aureus* (VRSA) [44]. Moreover, most previous reports have focused on single biological activities without providing a comprehensive evaluation that integrates antibacterial, antibiofilm, and antibiotic-potentiating effects alongside cytotoxicity profiling. In addition, concerns related to the cytotoxicity of monometallic silver nanoparticles toward normal cells are still not fully resolved, and the potential role of selenium incorporation in modulating this toxicity requires further investigation [15]. Furthermore, the utilization of agro-waste-derived extracts as sustainable reducing agents for the synthesis of such multifunctional nanocomposites remains insufficiently explored. Therefore, there is a clear need for developing eco-friendly synthesized bimetallic nanocomposites with enhanced efficacy, reduced toxicity, and multifunctional biomedical applications [45].

According to the previous background, the current study aimed to biologically synthesize the bimetallic Ag–Se NC using *C. melo* L. peel extract. Additionally, evaluate their cytotoxicity using various cell lines. Also, its different biological effects, including the antibacterial activities against VRSA clinical isolates, were estimated.

## 2 Materials and methods

### 2.1 Materials

Silver nitrate (AgNO₃, ≥ 99.9% purity) and sodium selenite (Na₂SeO₃, ≥ 99% purity) were purchased from Sigma-Aldrich (St. Louis, MO, USA) and used without further purification. All other chemicals and reagents were of analytical grade and obtained from the same supplier unless otherwise stated. Microbiological culture media, including Mueller–Hinton agar (MHA), Mueller–Hinton broth (MHB), Luria–Bertani broth (LB), and nutrient broth, were obtained from HiMedia (Mumbai, India). The WI-38 normal and cancerous cell lines, including Hep-G2 and MCF-7, were obtained from the American Type Culture Collection (ATCC). In addition, double-distilled water (ddH₂O) was employed in the current work.

### 2.2 Preparation of *C. melo* L. peel extract

The peel of melon fruit (*Cucumis melo* L.) was collected from New Valley Governorate, Egypt. *Cucumis melo* L. was identified by Prof. Dr. Abdou Marie Hamed at Ecology Lab., Botany and Microbiology Dep., Faculty of Science, Al-Azhar University, Cairo, Egypt. Experimental research and field studies involving plants, including the gathering of plant specimens, adhere to applicable institutional, national, and international regulations and legislation.

*Cucumis melo* L. was washed thoroughly with tap water to remove dust and debris, and rinsed with double-distilled water (ddH₂O). The peels were air-dried until constant weight and ground into a fine powder. A total of 20 g of the powder was mixed with 300 mL of ddH₂O and subjected to reflux extraction at approximately 100 °C for 60 min in a 500 mL round-bottom flask. The extract was allowed to cool to room temperature and filtered through Whatman No. 1 filter paper (pore size 11 µm). The dried extract weight was measured, yielding a 25% extraction yield. The filtrate was collected and stored at 4 °C in airtight containers until further use. The phytochemical composition, including total phenolics, flavonoids, and major compounds such as 3-hydroxybenzoic acid and apigenin-7-glycoside, has been previously reported and is referenced accordingly [46–49].

### 2.3 Preparation of Ag–Se NC

The green synthesis of Ag–Se NCs was carried out by preparing separate 5 mM aqueous solutions of Na₂SeO₃ and AgNO₃. The selected equimolar solutions were determined through preliminary optimization experiments to achieve the best formation of the Ag–Se nanocomposite. A volume of 20 mL from each precursor solution was mixed with 60 mL of C. melo peel extract under continuous magnetic stirring, resulting in a total reaction volume of 100 mL. The reaction was conducted in open air at 60 °C with constant stirring at 500 rpm. The pH of the extract was initially measured at 6.8 and then carefully adjusted to 9 using 1 N NaOH, with continuous monitoring by a calibrated pH meter. The reaction mixture was maintained for 2 h, during which a visible color change indicated the formation of Ag–Se NCs. The product was collected by centrifugation at 10,000 rpm for 15 min, washed three times with double-distilled water to remove unreacted residues, and dried in an oven at 100 °C for 3 h under air atmosphere [50]. The dried Ag–Se NCs were stored in airtight glass vials at room temperature (25 °C) and protected from light until further use.

The yield of the Ag–Se nanocomposites was calculated as the mass of dried product obtained after centrifugation, washing, and drying, relative to the total mass of AgNO₃ and Na₂SeO₃ precursors. The yield was expressed as a percentage using the formula:


$$\begin{array}{c} \text{Yield }(\%)=\text{Mass of dried Ag–Se NCs obtained }(\text{g})/\text{Mass of precursors }(\text{g})\times \text{100} \end{array}$$

Based on this calculation, the yield of the Ag–Se nanocomposites was determined as the mass of the dried product obtained after centrifugation, washing, and drying, relative to the total mass of AgNO₃ and Na₂SeO₃ precursors. The dried product weighed 0.025 g, corresponding to an estimated yield of 89%. This provides an estimate of the reaction efficiency under the optimized conditions.

### 2.4 Characterization of Ag–Se NC

The formation of Ag–Se NCs was preliminarily indicated by a visible color change in the *C. melo* L. peel extract, transitioning from light yellow to brownish-black. UV–visible spectroscopy was conducted in the range of 200–700 nm using a UV–Vis spectroscopy, with a scan rate of 200 nm/min and a slit width of 2 nm to monitor the surface plasmon resonance (SPR) of the synthesized nanocomposites. Fourier transform infrared (FTIR) spectroscopy was performed within the range of 400–4000 cm ⁻ ¹ using the KBr pellet method, with a resolution of 4 cm ⁻ ¹ and 32 scans per sample, to identify the functional groups responsible for the reduction and stabilization of the NCs. For TEM analysis, a small amount of the dried Ag–Se nanocomposites was dispersed in ethanol by ultrasonication for 10 min to ensure uniform dispersion. A drop of the suspension was placed onto a carbon-coated copper grid and allowed to dry at room temperature before imaging. The morphology and particle size were examined using transmission electron microscopy (TEM, JEM-2100 Plus) operated at an accelerating voltage of 200 kV.

Hydrodynamic size distribution was determined by dynamic light scattering (DLS) using a Nano ZS instrument (Malvern Instruments, UK) at 25 °C. Three consecutive measurements were performed for each sample to ensure reproducibility. Zeta potential (ζ) measurements were also carried out to evaluate the surface charge and colloidal stability of the samples. For further characterization, a small amount of the dried Ag–Se nanocomposites was dispersed in double-distilled water by ultrasonication for 10 min to obtain a uniform suspension for TEM and DLS analyses. For XRD analysis, the dried Ag–Se nanocomposites were finely ground using an agate mortar and pestle to obtain a homogeneous powder. The powder was evenly spread on a glass sample holder and gently pressed to ensure a flat surface for diffraction measurements. The crystalline structure and average crystallite size were analyzed using X-ray diffraction (XRD, XRD-6000, Shimadzu) with Cu Kα radiation (λ = 1.5406 Å), over a 2θ range of 10–90°, with a step size of 0.02° and a scanning rate of 2°/min. The average crystallite size of the Ag–Se nanocomposites was estimated using the Scherrer equation:


$$\begin{array}{c} D=K\lambda /\beta \text{cos}\theta \end{array}$$

where 𝐷 is the crystallite size, 𝐾 is the shape factor (0.9), λ is the X-ray wavelength (1.5406 Å), β is the full width at half maximum (FWHM) of the diffraction peak in radians, and θ is the Bragg angle. Scanning electron microscope (SEM) imaging (SEM, ZEISS, EVO-MA10, Germany) revealed surface morphology. Finally, the ZnO-MnO NCsformulation elements and their distribution were examined withspectroscopy based on the energy-dispersive X-ray (EDX) analysis (EDX, Bruker, Germany).

### 2.5 Cytotoxicity of Ag–Se NC toward normal and cancerous cell lines

The WI-38 normal cell line (ATCC CCL-75) was used for evaluation of Ag–Se NC (500–15.62 µg/mL) cytotoxic effect based on the 3-(4,5-dimethylthiazol-2-yl)-2,5-diphenyltetrazolium bromide (MTT) method [51]. Also, the malignant proliferation inhibitory effect was assessed towards the Hep-G2 (ATCC HB-8065) and MCF-7 (ATCC HTB-22) cancerous cell lines at different concentration ranged from 250 to 7.81 µg/mL. A 96-well tissue culture plate was inoculated with a cell suspension of 1 × 10^5^ cells/mL, with 100 µL dispensed into each well. After plates were incubated for 24 hrs at 37°C, the growth medium was carefully aspirated, the cell monolayer was washed twice using washing medium, and two-fold dilutions of the test sample was prepared in RPMI maintenance medium supplemented with 2% serum. A total of 0.1 mL of each dilution was then dispensed into designated wells, while three wells were reserved as controls, receiving only the maintenance medium. Following the plates’ incubation at 37°C for 24 h, the cell viability was evaluated through inoculation of 20 µL MTT solution prepared to a final concentration of 5 mg/mL using phosphate-buffered saline (PBS). After thoroughly mixing via 5 min shaking at 150 rpm, the plates were incubated in the presence of 5% CO_₂_ in a 37°C atmosphere for 4 hrs. The optical density (OD) at approximately 560 nm (OD560) of the tested sample and control was then determined with three replicates. The final total cell count was adopted to calculate the ratio of viable cells according to the following equations.


$$\begin{array}{c} \text{Viability }\%=\;\frac{\text{Sample OD560}}{\text{Control OD560}}\text{ X 100} \end{array}$$

$$\begin{array}{c} \text{Cytotoxicity }\%=\text{100}-\text{Viability }\% \end{array}$$

The half-maximal inhibitory concentration (IC₅₀) values were calculated using nonlinear regression analysis by fitting the dose–response data to a sigmoidal curve based on the Hill equation. This approach provides a more accurate estimation of IC₅₀ compared to linear methods, as it accounts for the full dynamic range and slope of the response curve.

Furthermore, the selectivity toward cancer cells can be evaluated using the Selectivity Index (SI), defined as the ratio of IC₅₀ in normal cells to IC₅₀ in cancer cells, as shown below:


$$\begin{array}{c} SI=\frac{IC_{\text{5}\text{0}\;(normal\;cells)}}{IC_{\text{5}\text{0}\;(cancer\;cells)}} \end{array}$$

### 2.6 Antibacterial assays

#### 2.6.1 The tested isolates.

A total of phenotypically identified 42 *S. aureus* clinical isolates, with different capabilities for biofilm formation, from our previous study [52], were included in the present study. In all assays, the tested bacterial isolate suspension was prepared using pure and fresh colonies at a concentration of 1.5 X 10^8^ CFU/mL that is equivalent to 0.5 McFarland standard turbidity.

Broth microdilution assay guidelines recommended by the Clinical Laboratory Standard Institute [53]—Performance Standards for Antimicrobial Susceptibility Testing, 34th Edition, M100—were conducted for evaluation of vancomycin susceptibility, applying a 2-fold serial dilution with a concentration range of 512−1 µg/mL. Consequently, *S. aureus* isolates exhibiting a vancomycin minimum inhibitory concentration (MIC) equal to or more than 32 µg/mL were recognized as VRSA strains [54].

#### 2.6.2 Inhibitory effect using agar well diffusion assay.

The preliminary screening for biogenic Ag–Se NC activity against VRSA clinical isolates was examined based on the agar well diffusion [55] assay [52]. In brief, the pure bacterial suspension was aseptically inoculated on MHA to produce lawn microbial growth. Equidistance of 6 wells of 6 mm diameter each were then made on the MHA surface, followed by the addition of few drops of melted MHA to each well to prevent the escaping of tested materials. The suspension (80 µL) of Ag–Se NC in dimethyl sulfoxide (DMSO) of 1000 µg/mL was then inoculated to the 1^st^ well, while the 2^nd^ well was inoculated with 80 µL of linezolid (30 µg/mL), which acts as a positive control. The negative control tests were included in the remaining wells that were separately inoculated with 80 µL of the solvent (DMSO 10%), *C. melo* L. peel extract, or the precursors used for NC preparation, including silver nitrate (1000 µg/mL) and sodium selenite (1000 µg/mL). The experiment plates were consequently reserved in the refrigerator for 30 min to permit the pre-diffusion of the tested suspensions into the inoculated agar. Following aerobic incubation for 24 hrs at 37°C, each inhibition zone diameter (IZD) correlated to the potential activity of the tested materials was measured. The experiment was conducted in triplicate (n = 3), and the mean ± standard deviation (SD) was used for presentation of the assay findings. The potential activity of the tested Ag–Se NC was correlated to that of linezolid via calculation of the percentage inhibition (PI) according to the following equation.


$$\begin{array}{c} \text{Percentage Inhibition }(\text{PI})=\;\frac{\text{IZD caused by the tested NC}}{\text{IZD caused by the positive control}}\text{ X }\text{1}\text{0}\text{0} \end{array}$$

#### 2.6.3 Inhibitory effect using broth microdilution assay.

The quantitative broth microdilution assay based on resazurin was applied to examine the potential capability of Ag–Se NC to terminate the tested VRSA clinical isolates’ growth. Also, its in-vitro bactericidal or bacteriostatic behavior was evaluated [56]. Concisely, the biogenic Ag–Se NC was 2-fold serially diluted in each microtiter plate row using MHB, in a range of 512–8 µg/mL, followed by the inoculation of the tested bacterium. The included positive control test contained MHB and tested bacterium without Ag–Se NC, while the negative control test contained MHB medium and Ag–Se NC with no bacterial inoculum. After the incubation for 24 hrs at 37°C, 20 µL of resazurin (0.015%) was added into each well with a consequent incubation at 37°C for 2 hrs. The well showed no resazurin color change from blue to red in the presence of the lowest Ag–Se NC concentration, which was recorded as the MIC. The experiment was conducted in triplicate (n = 3). For determination of minimum bactericidal concentration (MBC), bacterial inoculum from all wells containing ≥ MIC of the NC was cultured on MHA, followed by overnight incubation at 37°C. The lowest concentration of Ag–Se NC causing no viable microbial growth was scored as the MBC of the biosynthesized Ag–Se NC. Finally, the Ag–Se NC MIC index (MICi), or tolerance level, of each VRSA isolate was estimated according to the following equation.


$$\begin{array}{c} \text{Tolerance level}=\frac{\text{MBC}}{\text{MIC}} \end{array}$$

The tolerability of the Ag–Se NC of 4 or less correlated to its bactericidal effect, while the tolerability of more than 4 reflected its limited bacteriostatic ability against the tested VRSA clinical isolates [57,58].

The MIC for 50% (MIC_50_) was calculated after measurement of OD at 600 nm (OD_600_) that reflects the microbial growth [59]. The MIC_50_ for the entire tested population was calculated according to the following equation.


$$\begin{array}{c} {\text{MIC}}_{\text{5}\text{0}}=\text{C1}+\frac{\text{50}-\text{G1}}{\text{G2}-\text{G1}}\times (\text{C2}-\text{C1}) \end{array}$$

Where:

- C₁ = the lower concentration at which growth (G₁) is closest above 50%

- C₂ = the higher concentration at which growth (G₂) is closest below 50%

- G₁ and G₂ = the corresponding growth percentages at concentrations C₁ and C₂, respectively

#### 2.6.4 Antibiofilm assay.

##### 2.6.4.1 Biofilm inhibitory assay.

The ability of biogenic Ag–Se NC, at its 0.5 MIC for each tested isolate, to inhibit VRSA biofilms was studied following a crystal violet (CV) assay using a microtiter plate [60,61]. In brief, the tested biofilm-forming VRSA isolates were aseptically inoculated into sterile Luria-Bertani containing 2% glucose (LBG) broth. Following incubation at 37°C for 18 hrs, each tested bacterial suspension was diluted 1:100 using fresh LBG broth; 40 µL of bacterial suspension was added into each microtiter plate well followed by the addition of 10 µL of the tested Ag–Se NC (T). The assay was conducted in triplicate, and NC untreated well was used as a control (C). After incubation at 37°C for 24 hrs, the excess medium was discarded, and the bacterial biofilm was rinsed 3 times using PBS (0.1 M, pH 7.4). The CV (0.4%) solution was used to stain bacterial cells present in the adherent biofilm in the positive control and test experiments, followed by incubation at 37°C for 20 min. The redundant stain was then removed with subsequent washing using PBS three times, followed by solubilization of the CV remaining in each well using 200 µL ethanol (95%). To finish, OD590, reflecting the intensity of the formed biofilm, was examined, and the reduction percentage of bacterial biofilm caused by the NC was calculated based on the following biofilm reduction equation.

##### 2.6.4.2 Biofilm clearance assay.

The ability of the tested NC to eliminate the preformed VRSA biofilm was also assessed [54]. In brief, after incubation at 37°C for 48 hrs and careful discarding of the remaining LBG broth containing non-adherent planktonic bacterial cells, the established, fully formed VRSA biofilm was gently washed using PBS. Each tested VRSA strain biofilm in LBG broth was then treated with the biosynthesized Ag–Se NC at 0.5 MIC; an untreated positive control was included. Following 24 hrs of incubation at 37°C, the remaining LBG broth and NC were carefully removed, followed by PBS washing. The CV 0.1% solution was then used for 15 min at 37°C to stain the attached bacterial cells. Following PBS washing and air drying, 95% ethanol was employed for solubilization of the attached dye. The biofilm clearance percentage caused by the biosynthesized NC was assessed, after determination of the OD_590_, according to the biofilm clearance equation. The experiment was conducted in triplicate (n = 3), and the mean ± standard deviation (SD) was used for presentation of the assay findings.


$$\begin{array}{c} \text{Biofilm reduction or clearance }(\%)\;=\;\text{1}\;-\;\frac{\text{Mean OD590 of biofilm formed in }(\text{T})}{\text{Mean OD590 of biofilm formed in }(\text{C})}\text{ X }\text{1}\text{0}\text{0} \end{array}$$

### 2.7 Killing kinetics

The time-kill assay is widely employed for assessing NC-based therapeutics via providing of quantitative and time-resolved analysis of their antimicrobial efficacy. So, this assay was performed to evaluate the bactericidal activity of bimetallic Ag–Se NC against selected highly susceptible VRSA isolates using the viable count method [61,62]. The tested bacterial suspension, prepared in nutrient broth and adjusted to 10^6^ CFU/mL, was treated with Ag–Se NC in the form of 2-fold serial dilutions equivalent to 4 × MIC, 2 × MIC, and MIC. During incubation at 37°C, aliquots were taken at 0, 2, 4, 6, 8, and 24 hrs intervals with subsequent serial dilution using sterile saline, inoculation of each obtained dilution into nutrient agar, and incubation at 37°C for 24 hrs followed by enumeration of CFU, which presented as log₁₀ CFU/mL. A reduction of ≥ 3 log_10_ CFU/mL compared to the original inoculum was considered an indicator of bactericidal activity. The experiment was conducted in triplicate (n = 3), and the results are expressed as mean ± standard deviation. The lower limit of detection (LOD) of the viable plate count method was determined based on the plating volume and the lowest dilution factor employed. In this study, 100 µL aliquots of the lowest dilution were spread-plated onto nutrient agar, yielding a theoretical LOD of 10 CFU/mL (1.0 log₁₀ CFU/mL). Accordingly, bacterial counts reported as zero in the time-kill curves indicate that viable counts had fallen below this detection threshold rather than representing absolute eradication [63].

### 2.8 Protein leakage assay

This assay was conducted to evaluate the membrane-disrupting activity of Ag–Se NC against one selected highly susceptible VRSA isolate [64]. A fresh colony of the tested VRSA isolate was inoculated into MHB and cultivated at 37°C with shaking at 180 rpm until reaching the mid-exponential growth phase (OD_600_ ≃ 0.6). For removing residual broth components and extracellular proteins, bacterial cell harvesting was carried out at 4°C via centrifugation for 10 min at 8000 × g, followed by twice washing for the obtained pellet using sterile PBS. The obtained cell pellets were then suspended using PBS equivalent to a cell density of 1 × 10^8^ CFU/mL, confirmed by measuring OD_600_ and correlating with a predetermined standard curve. The obtained cell suspension (1 mL) was then transferred into a sterilized microcentrifuge tube (2 mL), followed by the addition of Ag–Se NC at final concentrations equivalent to 1/4x, 1/2x, 1x, and 2x of the predetermined MIC. A tube containing only PBS served as the negative control, representing baseline leakage without any lytic agent. A tube containing a 1% (v/v) solution of Triton X-100 served as the positive control, representing complete lysis and maximum protein release. All treated samples and controls were thoroughly mixed and subsequently incubated at 37°C for a period of 2 hrs to allow for membrane interaction and potential disruption. After that, the samples were immediately centrifuged at 12,000 × g for 15 min at 4°C to pellet any intact bacterial cells and cellular debris. After aspiration of the clarified supernatant from each tube, a Bradford protein assay was conducted to quantify its protein content. In a clean cuvette, the obtained supernatant (100 µL) was mixed with Bradford reagent (1 mL), followed by vertexing and subsequent standing for 10 min at room temperature and absorbance measurement spectrophotometrically at 595 nm. A blank sample containing PBS mixed with Bradford reagent was used. The background absorbance of Ag-Se NCs suspended in the assay medium (without bacterial cells) was measured and deducted from all related test results to account for any optical interference caused by the nanoparticles. The average absorbance value for each triplicated experiment was calculated, and the protein leakage percentage for the tested sample was estimated via comparison with the absorbance values of the negative and positive controls. The experiment was conducted in triplicate (n = 3), and the results are expressed as mean ± standard deviation.

Bovine serum albumin [65] was used as the protein standard to generate a calibration curve. In a series of BSA standard solutions (ranging from 0 to 1000 µg/mL), 100 µL of each standard or test sample was mixed with 1 mL of Bradford reagent (Coomassie Brilliant Blue G-250). After 5 minutes of room temperature incubation, the absorbance at 595 nm was measured using a UV-Vis spectrophotometer. The protein concentration in each sample was calculated by interpolating the BSA standard curve [66].

### 2.9 Synergistic antibacterial assay

Checkerboard titrations, using the resazurin-based broth microdilution method in a microtiter plate, were applied to evaluate the possible synergistic interaction between the biogenic Ag–Se NC when combined with vancomycin, at different concentrations, against 11 VRSA clinical isolates [67]. In brief, the assay depends on 2-fold dilution, using Luria-Bertani broth, of vancomycin along the X-axis, in the range of 0.5 MIC-0.015 MIC [68], using equal volumes in the plate’s columns, while the rows contained the tested NC serially diluted at 2-fold along the Y-axis, in the range of 0.5 MIC-0.015 MIC, with equal volumes. This assay design resulted in a unique combination between the vancomycin and Ag–Se NC in each well. After 24 hrs of incubation at 37°C, resazurin dye was used for the determination of the MIC for each row. Finally, the fractional inhibitory concentration (FIC) and the FICi for each established MIC were calculated using the following equations.


$$\begin{array}{c} \text{FIC }=\;\frac{\text{MIC of the tested compound in the used combination}}{\text{MIC of the tested compound when used alone}} \end{array}$$

$$\begin{array}{c} \text{FICi}=\text{FICAg}-\text{Se NC }+\text{ FICvancomycin} \end{array}$$

The calculated FICi < 0.5 refers to the synergistic Ag–Se NC and vancomycin interaction, while the FICi equal to 0.5–0.75 signifies their partial synergistic interaction, and FICi equal to 0.76–1.0 illustrates their additive effect. Conversely, the FICi > 1.0 but ≤ 4.0 indicates the indifferent effect, while the FICi > 4.0 indicates the undesirable antagonistic effect. Additionally, the experiments were performed in triplicate, and the results are reported as mean ± SD. Statistical analysis was performed using a paired t-test, and differences were considered statistically significant at p < 0.05.

### 2.10 Statistical analysis

GraphPad Prism v8 (GraphPad Software, San Diego, CA, USA) was used for all statistical analysis and figure preparation. Each experiment was conducted in triplicate (n = 3). The results are displayed as the mean ± the standard deviation (SD), and. Before performing any parametric tests, the Shapiro-Wilk test was employed to assess data normality. For the agar well diffusion assay, a paired sample t-test was used to compare the inhibitory zone diameters of Ag-Se NC with the linezolid positive control. In the broth microdilution experiment, one-way ANOVA and Tukey’s post hoc test were used. When the normality assumption was not met, the non-parametric Kruskal-Wallis test was used in its place. For the antibiofilm experiments, one-way ANOVA and Tukey’s post hoc test were used to evaluate the percentages of biofilm inhibition and clearance. Pearson’s correlation coefficient was calculated to evaluate the relationship between biofilm inhibition and clearance activities. For the time-kill test, two-way ANOVA was employed. For the protein leakage experiment, two-way ANOVA was used to evaluate the concentration-dependent effect of Ag Se NC on membrane rupture. Pearson’s correlation coefficient was calculated to assess the relationship between NC concentration and protein leakage. To classify the interaction between Ag Se NC and vancomycin, the fractional inhibitory concentration index (FICi) for each isolate was calculated using the checkerboard synergy assay. A paired t-test was used to compare the MIC values of each medication alone and in combination. Additionally, Combenefit^®^ software was used to construct synergy/antagonism maps based on various analytical models (Highest Single Agent, Bliss Independence, and Loewe Additivity). In all analyses, *P* ≤ *0.05*0.05 was considered statistically significant.

## 3 Results and discussion

### 3.1 Preparation of Ag–Se NC

The biosynthesis of Ag–Se NC was successfully achieved using the aqueous peel extract of *C. melo* L., which functioned as both a reducing and stabilizing medium. Upon the addition of silver nitrate and sodium selenite to the plant extract, a rapid color change from light yellow to brownish-black was observed. This visible transition confirms the progression of reduction reactions and the formation of Ag–Se nanocomposites, and it is attributed to surface plasmon resonance (SPR) arising from the generated nanoscale structures rather than simple aggregation. The formation mechanism can be explained through sequential steps. Initially, bioactive phytochemicals present in the peel extract, particularly phenolic compounds, flavonoids, and reducing sugars, interact with Ag⁺ and Se⁴ ⁺ ions through complexation and electrostatic attraction [69]. These biomolecules subsequently act as electron donors, facilitating the reduction of Ag⁺ to Ag⁰ and Se⁴⁺ to Se⁰ [70,71]. The reduced atoms then undergo nucleation, leading to the formation of initial Ag and Se nuclei. Continuous growth of these nuclei results in the formation of Ag–Se nanocomposite structures, where both components coexist at the nanoscale. Finally, residual phytochemicals adsorb onto the nanoparticle surface, acting as capping agents that stabilize the formed nanocomposites and prevent aggregation through steric and electrostatic repulsion. The melon revealed many phytochemicals comprising phenolic compounds, flavonoids, and reducing sugars that are capable of reduction of the metal ions and provided stability to the NC [72]. This visible color transformation served as a primary indicator of NPs formation, which was further supported by subsequent characterization analyses. Mohan et al. [73] reported the green synthesis of silver–palladium (Ag/Pd) BNCs using *Catharanthus* leaf extract as a natural reducing and stabilizing agent. Idris et al. [74] reported the eco-friendly synthesis of silver oxide–nickel oxide (Ag_2_O–NiO) BNCs using discarded orange peels (*Citrus sinensis* (L.) Osbeck) for the natural NC reduction and stabilization process. Sher et al. [75] synthesized silver–gold (Ag/Au) BNCs using *Hippeastrum hybridum* (HH) extract as a biogenic reducing and stabilizing agent. Khan et al. [76] reported the green synthesis of silver–copper (Ag–Cu) BNCs using *Peganum harmala* leaf extract as a natural reducing and stabilizing agent. Rani et al. [77] synthesized Ag NPs using *C. melo* L. leaf extract through a green synthesis method and evaluated their potential against diabetes and coccidiosis under *in vitro* conditions. Kang et al. [78] recorded the enhanced *C. melo* L. plant’s photosynthetic activity after treatment with Se NPs.

The successful formation of Ag–Se NC can be attributed to the synergistic role of the extract constituents, which simultaneously enable reduction and stabilization processes under the applied reaction conditions. Parameters such as extract composition, temperature, and pH collectively influence the nucleation rate, particle growth, and final stability of the nanocomposites, leading to the formation of well-dispersed and stable Ag–Se NC.

### 3.2 Characterization of Ag–Se NC

The biosynthesized Ag–Se NC obtained using the aqueous peel extract of *C. melo* L. was subjected to comprehensive physicochemical characterization to confirm its formation, composition, morphology, and crystalline nature. A range of analytical assessments, encompassing UV–vis spectroscopy, FTIR, TEM, DLS, and XRD techniques, were employed to offer unique insights about the optical and structural characters of the NC. The spectroscopic analyses confirmed the reduction of metal ions and the successful formation of the Ag–Se NC, while microscopic and diffraction analyses revealed details regarding particle size, shape, crystallinity, and elemental distribution.

### 3.3 UV–vis spectroscopy

**Fig 1:**
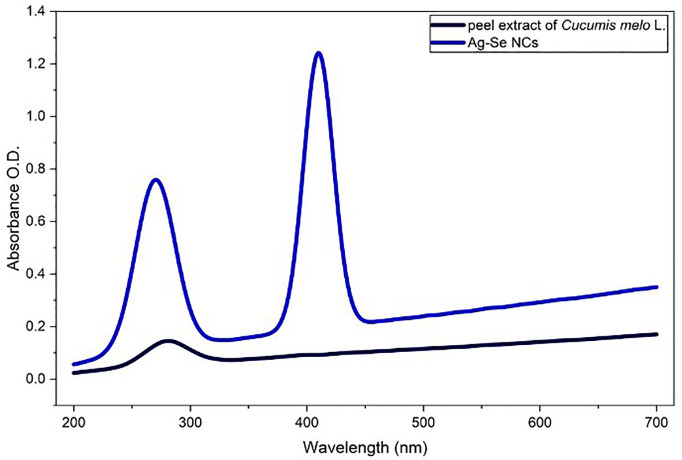
UV–Vis absorption spectra of *Cucumis melo* L. peel extract and the biosynthesized Ag–Se NC, showing the optical properties of the plant extract and the synthesized nanocomposite.

The UV–Vis absorption spectra of the *C. melo* L. peel extract and the biogenic Ag–Se nanocomposites are shown in Fig 1. The crude peel extract exhibits a very weak and nearly featureless absorption curve, indicating the absence of strong chromophoric or plasmonic species [72]. In contrast, the Ag–Se nanocomposite displays two distinct absorption peaks at 270 nm and 410 nm. The 270 nm peak corresponds to the characteristic electronic transition of Se nanoparticles, slightly shifted from the typical 250–350 nm range, likely due to interaction with Ag and capping phytochemicals. The 410 nm peak represents the surface plasmon resonance of Ag nanoparticles, influenced by particle size, shape, and the local dielectric environment provided by Se and the peel extract. Baseline correction and subtraction of the extract spectrum were applied, confirming that these peaks arise exclusively from the nanocomposite. The negligible absorption of the extract alone verifies that the observed optical features are primarily due to nanoparticle formation rather than phytochemicals [79,80]. Alafaleq et al. [81] reported the biogenic synthesis of Cu–Mn BNCs using pumpkin seed extract, with SPR band maxima observed at 266 nm and 336 nm. Ehsan et al. [82] reported that *Moringa oleifera*–mediated Ag–ZnO BNCs exhibited a broad absorption band with two shoulder peaks at 330 nm and 366 nm, confirming their successful formation. In contrast, Khan et al. [76] observed that Ag–Cu BNCs exhibited a distinct SPR peak at 555 nm. Furthermore, Hashem et al. [70] described the watermelon rind–mediated biosynthesis of bimetallic Se–Ag NPs, with the UV–vis absorption spectrum showing a characteristic peak at 380 nm. Rani et al. [77] established the successful formation of Ag NPs based on the UV–visible spectroscopic analyses, which showed a characteristic absorption peak at 440 nm. Hashem and Salem [83] reported the biosynthesis of Se NPs using Urtica dioica (stinging nettle) leaf extract, where the greatest absorption peak of the produced Se NPs was seen at 376 nm. Although the optical band gap of the synthesized nanocomposites can be estimated using Tauc plot analysis, this was not performed in the present study, as the UV–Vis measurements were primarily conducted to confirm nanoparticle formation. Further detailed optical investigations are recommended for future work.

### 3.4 FTIR spectroscopy

**Fig 2:**
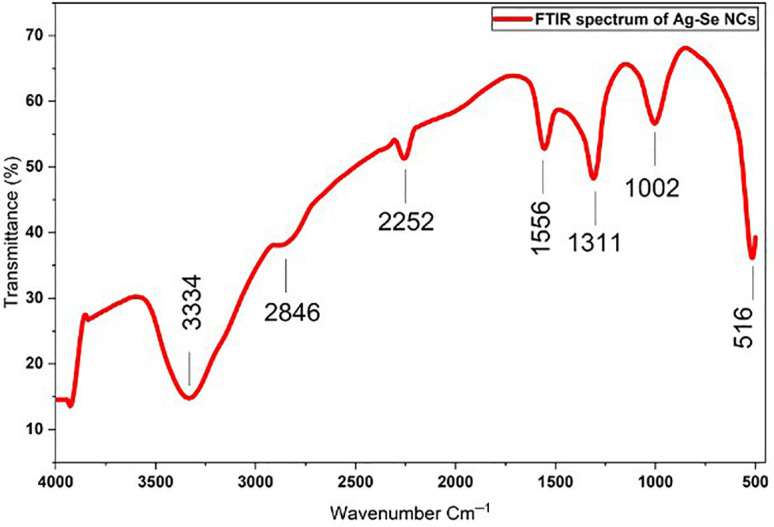
FTIR spectrum of the biosynthesized Ag–Se nanocomposite (Ag–Se NC), showing the characteristic absorption bands of functional groups associated with phytochemical constituents involved in the reduction, capping, and stabilization of the nanocomposite.

The FTIR spectrum of the biosynthesized Ag–Se NCs (Fig 2) revealed several characteristic absorption bands indicating the involvement of various functional groups from the peel extract of *C. melo* L. in the reduction and stabilization of the nanocomposites [79]. Notably, when compared with the spectrum of the crude plant extract, slight shifts in the position and intensity of several absorption bands were observed, suggesting the active participation of phytochemicals in the formation process through interaction with the nanoparticle surface.

The broad band at 3334 cm ⁻ ¹ corresponds to O–H stretching vibrations of hydroxyl groups, indicating phenolic and alcoholic compounds acting as reducing and capping agents [84]. The band at 2846 cm ⁻ ¹ is attributed to C–H stretching vibrations of aliphatic groups, while the absorption near 2252 cm ⁻ ¹ is assigned to C ≡ C or C ≡ N stretching vibrations of alkynes or nitriles [85,86]. Peaks at 1556 cm ⁻ ¹ and 1311 cm ⁻ ¹ correspond to N–H bending and C–N stretching vibrations, indicating the involvement of proteins or amine-containing biomolecules in stabilization [87,88]. The band at 1002 cm ⁻ ¹ is associated with C–O stretching vibrations of alcohols and ethers, confirming the role of polyphenolic compounds in the synthesis process [84].

Importantly, the band observed at 516 cm ⁻ ¹ may be attributed to metal–oxygen or metal–selenium vibrations, suggesting possible interaction between Ag and Se species. Importantly, the band observed at 516 cm ⁻ ¹ may be attributed to metal–O or metal–Se vibrations, which suggests the possible formation of Ag–Se nanocomposites; however, such assignment should be interpreted with caution, as FTIR alone cannot conclusively confirm metal–selenium bond formation [23]. However, this assignment should be interpreted cautiously, as FTIR analysis alone cannot conclusively confirm Ag–Se bond formation. Therefore, complementary techniques such as XPS or Raman spectroscopy would be required for definitive structural confirmation. Although the FTIR spectrum of the raw plant extract was not recorded for direct comparison, the identified functional groups are consistent with those typically reported in plant-derived extracts, supporting their role in the reduction and stabilization of the nanocomposites.

### 3.5 TEM imaging and DLS analysis

**Fig 3:**
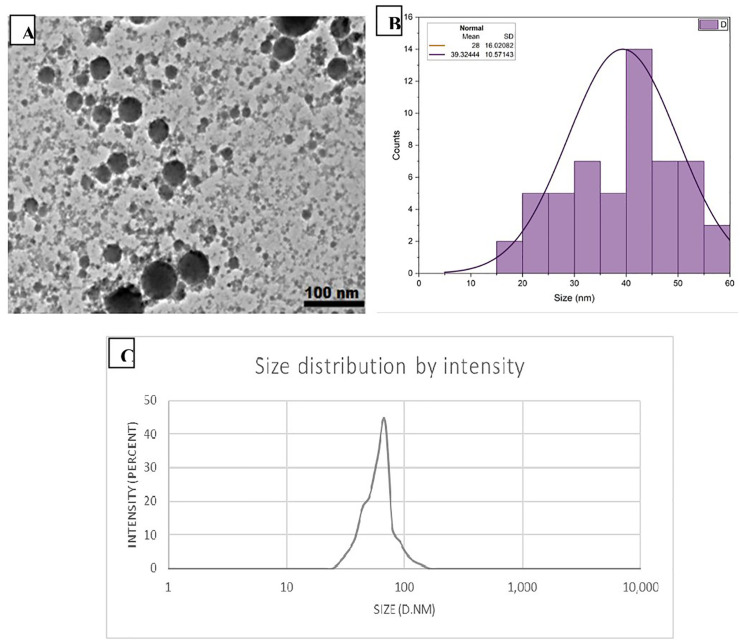
Transmission electron microscopy (TEM) image (A), particle size distribution histogram (B), and dynamic light scattering (DLS) analysis (C) of the biosynthesized Ag–Se NC.

Transmission electron microscopy (TEM) analysis (Fig 3A) was employed to investigate the morphology and size distribution of the biosynthesized nanocomposite (NC). The micrograph revealed that the NC predominantly exhibits a predominantly spherical morphology with relatively uniform distribution and minimal apparent agglomeration, as observed in the TEM micrograph. The particle size was found to range from 20 to 55 nm, with an average diameter of approximately 35 nm. The observed variation in particle size and morphology may be attributed to the complex biochemical environment provided by the plant extract, where multiple phytochemical constituents act simultaneously as reducing and stabilizing agents, influencing nucleation and growth processes. Fig 3B show particle size distribution histogram of the biosynthesized Ag–Se nanocomposite showing a relatively broad size range from approximately 15–60 nm. The distribution follows a near-normal pattern with a mean particle size of 39.3 nm and a standard deviation of 10.6 nm, indicating moderate polydispersity. The observed size distribution suggests the formation of nanoscale particles with slight aggregation tendencies, consistent with the SEM analysis.

Similar findings have been reported in previous studies on biosynthesized nanomaterials, where variations in particle shape and size were attributed to the heterogeneous nature of biological extracts and reaction conditions. For instance, Olawale et al. [89] reported Ag@Se nanobiocomposites synthesized using *Ocimum tenuiflorum* leaf extract with quasi-spherical and polyhedral morphologies and an average size of 33.1 nm, confirming effective bioreduction and stabilization. Similarly, San and Acar [90] observed Ag–Se nanoparticles with sizes ranging from 13.69 to 56.42 nm and an average diameter of 33.38 nm, while Mohan et al. [73] and Selim et al. [91] reported comparable nanoscale dimensions for Ag-based bimetallic systems, further supporting the consistency of biosynthesis approaches in controlling nanoparticle size within the nanometer range.

Dynamic light scattering (DLS) analysis was performed to evaluate the hydrodynamic size and colloidal behavior of the NC in aqueous suspension (Fig 3C). The results showed a hydrodynamic diameter ranging from 35 to 70 nm, which is larger than the particle size obtained from TEM. This difference is expected due to the fact that DLS measures the hydrodynamic diameter, which includes the solvent layer, surface-adsorbed biomolecules, and any weak interparticle interactions in the dispersed state, whereas TEM provides the dry core size of the nanoparticles. The polydispersity index (PDI = 0.159) indicates a narrow size distribution and suggests a relatively homogeneous colloidal system. This low PDI value reflects good control over nanoparticle growth during the biosynthesis process and effective stabilization by phytochemical constituents present in the plant extract. Overall, the combined TEM and DLS results suggest a relatively homogeneous and narrowly distributed nanoscale system.

Therefore, the NC particle sizes observed using DLS are mostly larger than those obtained via TEM imaging, yet they offer valuable information regarding particle stability and uniformity in suspension. Elkady et al. [92] reported that the bimetallic copper oxide–Se NPs synthesized using the leaf extract of *Lagenaria siceraria* exhibited an average particle size of approximately 79 nm, confirming their nanoscale formation and uniform distribution. Hashem et al. [70] conducted DLS analysis to evaluate the particle size distribution of the biosynthesized Se–Ag BNCs, revealing an average hydrodynamic diameter of 47.3 nm, which confirmed their nanoscale dimensions and good dispersion stability. More et al. [93] demonstrated that bimetallic Ag–Pt NPs synthesized via a green approach using *Ocimum basilicum* exhibited an average hydrodynamic size of about 59 nm, as determined by DLS analysis.

It is important to note that while the present results indicate good short-term colloidal stability, detailed long-term stability studies (e.g., time-dependent size distribution and zeta potential analysis) are required to fully elucidate the aggregation behavior and temporal stability of the nanocomposite system in different storage conditions. The low PDI value (0.159) further supports a relatively narrow size distribution and indicates a reasonably uniform dispersion in the colloidal system.

### 3.6 XRD analysis

**Fig 4:**
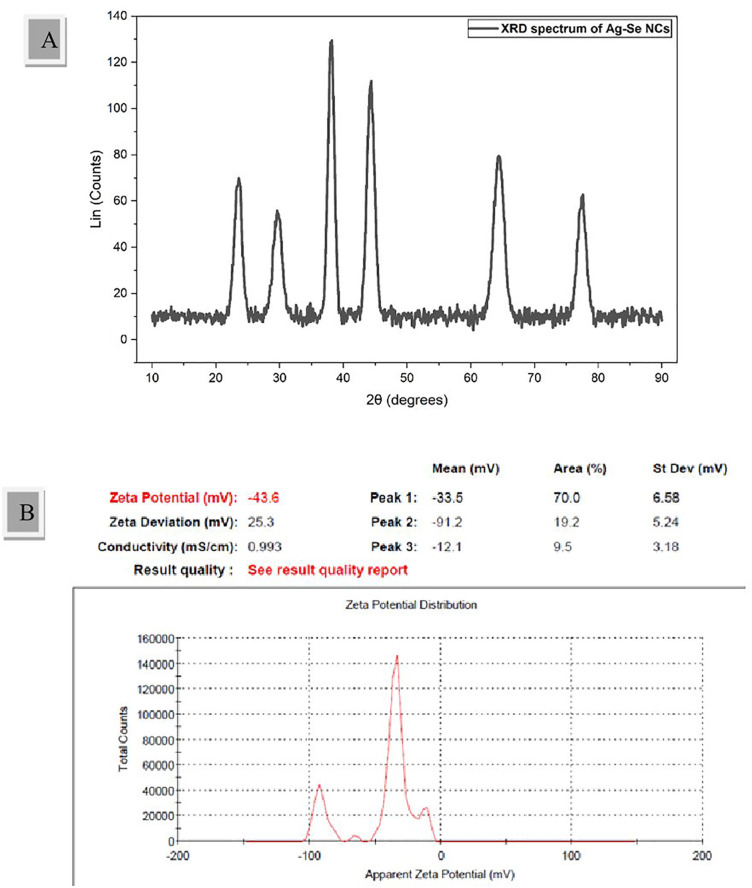
X-ay diffraction (XRD) pattern (A) showing the crystalline structure and zeta potential analysis (B) indicating the surface charge of the biosynthesized Ag–Se NC.

The XRD diffractogram of the biosynthesized Ag–Se NC (Fig 4A) showed definite and intense diffraction peaks detected approximately at 2θ = 27.6°, 32.1°, 38.2°, 44.3°, 64.4°, and 77.5°, demonstrating the crystallinity of the obtained NC. Also, the specific reflections at 38.2°, 44.3°, 64.4°, and 77.5° resemble the (111), (200), (220), and (311) planes of face-centered cubic (fcc) silver, in good accordance with the JCPDS standard card No. 04-0783 [94,95]. Rani et al. [77] reported that the XRD pattern of *C. melo*–mediated Ag NPs exhibited distinct and strong diffraction peaks, proving the synthesis of high-quality nanocrystals with a dissimilar crystalline nature, indicative of the successful biosynthesis and crystallinity of the NC. The XRD diffraction peaks of the biosynthesized Ag–Se NC were indexed according to their corresponding (hkl) planes, and the associated JCPDS card numbers are summarized in Table 1.

**Table 1:** Detailed assignment of XRD diffraction peaks of biosynthesized Ag–Se NC with corresponding (hkl) planes and JCPDS card numbers.

2θ (°)	hkl	Phase	JCPDS
27.6	101	Se	06-0362
32.1	102	Se	06-0362
38.2	111	Ag	04-0783
44.3	200	Ag	04-0783
64.4	220	Ag	04-0783
77.5	311	Ag	04-0783

Meanwhile, the other peaks at about 27.6° and 32.1° can be correlated to the (101) and (102) planes of hexagonal Se, matching well with JCPDS card no. 06-0362 [96,97]. These results are reliable with those of Fouda et al. [98], who recorded comparable diffraction peaks corresponding to the crystalline Se NPs. The coexistence of both sets of reflections confirms the successful formation of bimetallic Ag–Se NC without the presence of secondary impurities. The observed sharp and narrow diffraction peaks propose a well-defined NPs structure and high degree of crystallinity. Similar XRD patterns have been reported for biosynthesized Ag–Se nanomaterials by San and Acar [90] and Olawale et al. [89], who also observed characteristic reflections consistent with both Ag and Se crystalline phases. The slight peak broadening observed in the current spectrum may be attributed to nanocrystal size reduction and lattice strain resulting from the green biosynthetic procedures.

The biogenic Ag–Se NC crystallite size was calculated using the Scherrer equation: 𝐷 = 𝐾𝜆𝛽cos𝜃 where D is the average crystallite size (nm), 𝐾 is the shape factor (0.9), λ represents the Cu Kα radiation wavelength (0.15406 nm), 𝛽 is the full width at half maximum (FWHM) in radians, and θ denotes the Bragg diffraction angle. According to the broadening of the diffraction peak, the calculated crystallite size was 34 nm. This finding illustrates the formation of well-crystallized NPs. The observed average crystallite size is consistent with the TEM analysis, which also revealed nearly spherical particles within a similar size range. The diffraction peaks exhibited noticeable sharpness with slight broadening, indicating the formation of highly crystalline nanostructures with nanoscale crystallite size. The observed peak broadening may be attributed to the small particle size and possible lattice strain effects, which are typical characteristics of nanomaterials.

### 3.7 Zeta potential activity

Fig 4B illustrates the zeta potential distribution of the synthesized Ag–Se NC, showing an average zeta potential value of −43.6 mV. This highly negative value indicates the presence of strong electrostatic repulsion among the NC, which plays a significant role in preventing particle aggregation and maintaining colloidal stability in the aqueous medium. The zeta potential distribution curve exhibited three major peaks at −33.5 mV, −91.2 mV, and −12.1 mV with relative distributions of 70.0%, 19.2%, and 9.5%, respectively. The dominant peak at −33.5 mV suggests that the majority of NC possess a relatively uniform negative surface charge, reflecting a good degree of surface homogeneity. Meanwhile, the secondary peaks may indicate the presence of different surface functional groups derived from the phytochemicals involved in the green synthesis process.

Furthermore, the standard deviation values ranged from 3.18 to 6.58 mV, indicating relatively stable surface charge distribution among the nanoparticles. Overall, the zeta potential findings confirm that the synthesized NC exhibit good colloidal stability and low aggregation tendency, which may contribute to their enhanced biological activity. Zeta potential is considered one of the most important parameters for evaluating the stability of nanoparticles in liquid media [99]. Generally, zeta potential values exceeding ±30 mV are indicative of high colloidal stability due to strong electrostatic repulsion between particles [100]. In the present study, the synthesized NC exhibited a zeta potential value of −43.6 mV, confirming their high dispersion stability and reduced probability of aggregation or sedimentation during storage and biological applications. The observed high negative surface charge may be attributed to the adsorption of bioactive phytochemicals present in the plant extract used during the green synthesis process, such as phenolic compounds, flavonoids, and oxygen-containing functional groups [101]. These compounds act simultaneously as reducing and stabilizing agents, forming a protective layer around the nanoparticles and thereby enhancing their stability in aqueous environments [102].

In addition, the presence of multiple peaks in the zeta potential distribution suggests slight variations in surface functionalization or particle size distribution, which is commonly observed in biogenically synthesized nanoparticles [103]. Nevertheless, the predominance of the main peak representing 70% of the particle population reflects an acceptable degree of uniformity within the nanosystem. Doghish et al. [104] reported that the DLS data indicated a well-dispersed distribution of the synthesized Au NPs, with more than 75% of the particles exhibiting a uniform size distribution across all samples. Moreover, with increasing incubation time, the ζ-potential values stabilized at approximately −3.16 mV, suggesting a change in surface charge behavior during nanoparticle formation. Hosny et al. [105] reported that Ag and Cu nanoparticles loaded onto the biochar surface exhibited an almost spherical morphology with particle sizes ranging from 25 to 45 nm. The measured zeta potential of −25.5 mV indicated good colloidal stability of the Ag–Cu/biochar nanocomposite. Jeevarathinam et al. [106] reported the zeta potential [107] values of CuO NPs, ZnO NPs, and CuO–ZnO nanocomposites (CZ-NCs), providing insight into their surface charge characteristics. The recorded ZP values were −42.6, −26.4, and −44.8 mV for CuO NPs, ZnO NPs, and CZ-NCs, respectively. These strongly negative values indicate significant electrostatic repulsion between particles, which prevents aggregation and enhances the colloidal stability of the systems. The obtained results support the hypothesis that the good colloidal stability of the synthesized NC may directly contribute to improved interaction with microbial or viral cells, thereby enhancing their biological efficiency and overall bioactivity.

**Fig 5:**
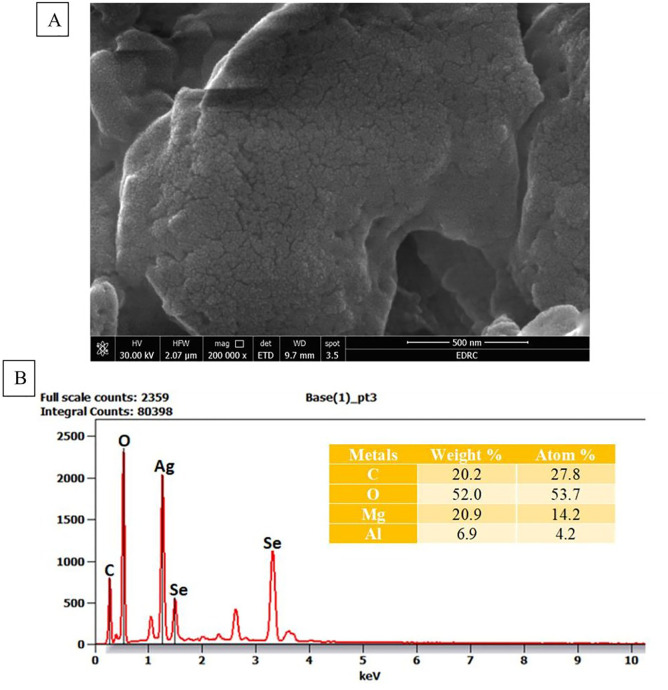
Morphological and elemental characterization of the biosynthesized Ag–Se NC using scanning electron microscopy (SEM) and energy-dispersive X-ray spectroscopy (EDX).

The SEM micrograph of the biosynthesized Ag–Se nanostructures (Fig 5A) reveals an irregular and highly aggregated morphology with a rough, granular surface texture. The observed structure consists of densely packed nanosized particles forming larger agglomerated clusters, which can be attributed to strong interparticle attractions and high surface energy associated with the biosynthesis process. The aggregates are mainly within the submicron range, indicating the successful formation of nanostructured materials. The image was acquired at a magnification of 200,000 × , enabling detailed observation of particle morphology and surface characteristics.

The EDX spectrum (Fig 5B) was used to determine the elemental composition of the synthesized sample. The analysis confirmed the presence of C, O, Ag, and Se elements, indicating their incorporation within the material. Oxygen showed the highest content (52.0 wt% and 53.7 at%), which may be attributed to surface oxidation of metallic species (Ag/Se) as well as oxygen-containing functional groups derived from phytochemical capping agents of the plant extract. Carbon (20.2 wt% and 27.8 at%) is likely associated with residual organic compounds from the plant extract and background carbon signals. Silver (Ag) was detected at 20.9 wt% (14.2 at%), while selenium (Se) was present at 6.9 wt% (4.2 at%), confirming their presence in the analyzed sample. Other study, Ulukuş et al. [108] performed EDS analysis to determine the elemental composition of Se@Ag/AgO, Se@ZnO, and Se@Ag/AgO–ZnO nanocomposites. The corresponding EDS spectra confirmed the presence and homogeneous distribution of Ag, O, Se, and Zn elements in all samples. While, for Se@Ag/AgO, the elemental composition was Ag (92.6%), O (7.1%), and Se (0.3%). In Se@ZnO, Zn, O, and Se were recorded at 77.7%, 22.0%, and 0.2%, respectively. Meanwhile, Se@Ag/AgO–ZnO nanocomposites contained Ag (52.2%), Zn (34.8%), O (12.6%), and Se (0.4%).

However, it is important to note that EDX provides only elemental composition and cannot alone confirm nanocomposite formation, chemical bonding, or interfacial interactions between the constituent elements. Therefore, the formation of Ag–Se nanostructures is further supported by complementary characterization techniques. The FTIR spectra confirmed the presence of functional groups from phytochemical constituents, which act as reducing and stabilizing agents and are involved in nanoparticle capping and surface modification [109]. In addition, XRD analysis demonstrated the crystalline nature of the synthesized material and confirmed the presence of characteristic diffraction peaks corresponding to Ag- and Se-related phases, indicating successful formation of crystalline nanostructures [110]. Collectively, SEM morphology, FTIR functional group analysis, and XRD crystallographic data provide strong evidence supporting the successful synthesis of Ag–Se nanostructured materials.

### 3.8 Correlation between physicochemical properties and biological activity

The biological performance of the synthesized Ag–Se nanocomposites can be directly correlated with their physicochemical characteristics. The nanoscale size (20–55 nm) and relatively narrow size distribution (PDI = 0.159) provide a high surface-to-volume ratio, facilitating enhanced interaction with bacterial cell membranes. This is consistent with the observed antibacterial activity, including low MIC values (64–512 µg/mL) and the rapid bactericidal effect demonstrated in time-kill assays, where complete eradication was achieved at 4 × MIC within 8 h. Moreover, the protein leakage assay confirmed a concentration-dependent disruption of membrane integrity, which can be directly attributed to the small particle size and high surface reactivity of the nanocomposites. These properties enable efficient attachment to the bacterial cell surface and subsequent membrane damage. The antibiofilm activity of Ag–Se NC can also be explained by their nanoscale dimensions and surface functional groups, which facilitate penetration into the biofilm matrix and interaction with embedded bacterial cells, resulting in both inhibition and partial eradication of established biofilms.

In terms of anticancer activity, the selective cytotoxicity observed toward MCF-7 and Hep-G2 cells compared to normal WI-38 cells may be attributed to enhanced cellular uptake and reactive oxygen species (ROS) generation induced by the Ag–Se system. The synergistic interaction between Ag and Se is likely to amplify oxidative stress in cancer cells, leading to apoptosis, while maintaining relatively lower toxicity toward normal cells. Collectively, these findings demonstrate that the physicochemical properties of Ag–Se nanocomposites play a critical role in governing their multifunctional biological performance.

### 3.9 Cytotoxicity of Ag–Se NC on normal and cancerous cell lines

**Fig 6:**
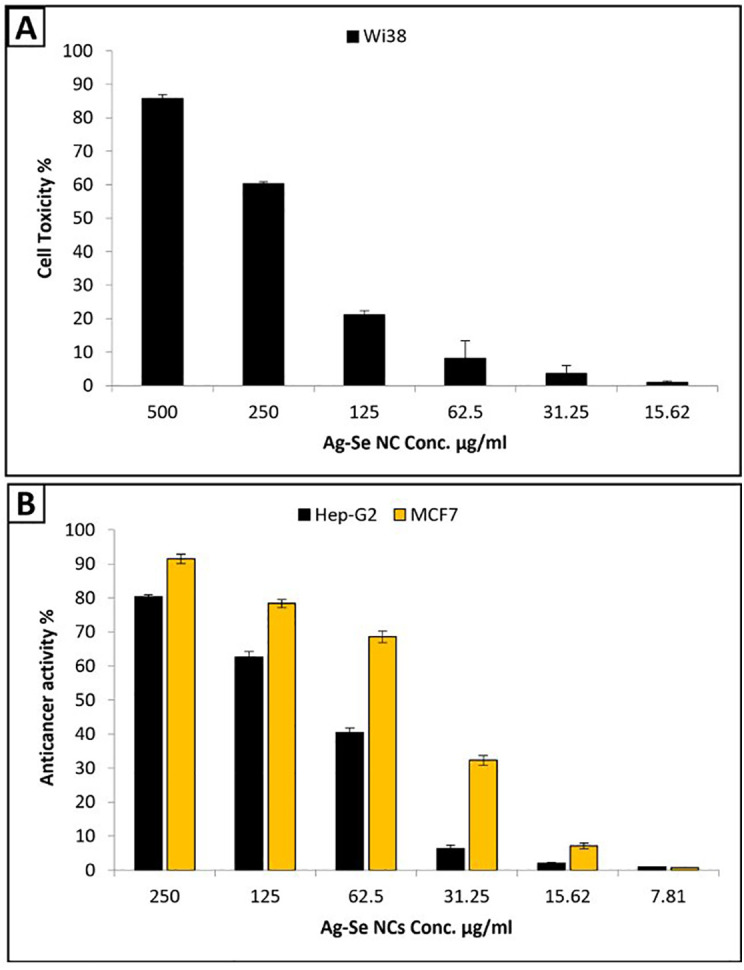
Evaluation of the cytotoxic activity of the biosynthesized Ag–Se NC against normal and cancer cell lines. **(A)** Cell viability of the WI-38 normal cell line following treatment with different concentrations of Ag–Se NC. **(B)** Cytotoxic effects of Ag–Se NC on MCF-7 and Hep-G2 cancer cell lines, demonstrating the concentration-dependent response of the treated cells.

In this study, a comprehensive evaluation of the dose-dependent cytotoxic and anticancer effects of Ag–Se NC on normal and malignant human cell lines clearly demonstrates their selective biological activity. In Fig 6A, the cytotoxicity of Ag–Se NC against WI-38 is presented as a percentage of cell toxicity among gradient doses ranging from 15.62 to 500 μg/mL. Results revealed that the IC_50_ of Ag–Se NC against WI-38 was 203.4 μg/mL. Generally, the IC_50_ value of 90 μg/mL or more indicates the classification of the tested compound as non-cytotoxic [111]. At 125 μg/mL, toxicity declines to around 20%, while at 62.5 μg/mL and below, cytotoxic effects become minimal (<10%), approaching negligible levels at the lowest tested concentrations. These findings highlight the biocompatibility of Ag–Se NC at low and moderate doses, suggesting that normal cells can tolerate therapeutically relevant concentrations with limited adverse effects.

In Fig 6B, the biogenic Ag–Se NC *in vitro* tumor inhibitory effect was evaluated against two cancerous cell lines, Hep-G2 and MCF-7, over NC concentrations in the range of 7.81–250 μg/mL. Both cancer cell lines exhibit a strong and dose-dependent reduction in viability following treatment with Ag–Se NC with IC50 90.97 and 38.18 μg/mL toward Hep-G2 and MCF-7 respectively. Notably, MCF-7 cells display consistently higher sensitivity compared with Hep-G2 cells at all tested concentrations. At 250 μg/mL, anticancer activity exceeds 90% in MCF-7 cells and reaches approximately 80% in Hep-G2 cells, indicating a potent inhibitory effect on cancer cell proliferation. Even at intermediate concentrations (125 and 62.5 μg/mL), Ag–Se NC maintains considerable anticancer efficacy, particularly against MCF-7 cells, where inhibition remains above 65%. In contrast, Hep-G2 cells show a more gradual decline in responsiveness, suggesting cell-type-specific susceptibility. At lower concentrations (≤15.62 μg/mL), anticancer activity is markedly reduced in both cell lines, indicating a threshold concentration is required for effective tumor cell inhibition. To confirm the safety of these compounds, the selectivity index (SI) was performed, where results showed that SI toward Hep-G2 and MCF-7 was 2.23 and 5.33 respectively. These results confirmed the safety of the both compounds, compounds possessing SI values greater than two are considered acceptable selectivity toward cancer cells.

Herein, Ag–Se NC possesses strong, concentration-dependent anticancer properties while exhibiting comparatively low toxicity toward normal cells at effective doses. The differential response between normal and cancerous cells underscores the selective cytotoxic potential of Ag–Se NC, supporting their promise as a nanomaterial-based anticancer agent with an improved therapeutic window.

The unique properties of these NPs enable them to produce ROS, which induces oxidative stress in cancer cells, leading to apoptosis via mitochondrial dysfunction and DNA damage. This mechanism is particularly effective in overcoming resistance seen in various cancers [112]. Additionally, functionalization of BNCs using specific targeting ligands allows selective interaction with specific receptors on the cancer cell. This targeted approach enhances the delivery of therapeutic agents directly to tumor sites, thereby increasing drug accumulation and minimizing systemic toxicity [113]. The synergistic interactions between the metals in BNCs can also improve their efficacy, making them more effective than their monometallic counterparts. Prior investigations have shown that BNCs display substantial anticancer properties. Utilizing the extract of *Salvia officinalis*, Asghari Moghaddam et al. [114] produced Ag–ZnO NC and found that the combined actions of Ag and ZnO increase the cytotoxicity against A549 lung cancer cells. A biosynthesized starch–Ag–Se NC with spherical/oval morphology (~68 nm) exhibited potent anticancer activity, notable antioxidant capacity, low hemolytic effect, and strong antimicrobial and antibiofilm activities [50]. Furthermore, the biogenic Se–Ag NC exhibited a strong anticancer effect towards the MCF-7 cancerous cell line, with a low IC_50_ value (21.6 µg/mL), indicating their strong therapeutic potential with acceptable safety towards normal cells [115].

### 3.10 Antibacterial assays

#### 3.10.1 Preliminary Ag–Se NC growth suppressive effect.

**Fig 7:**
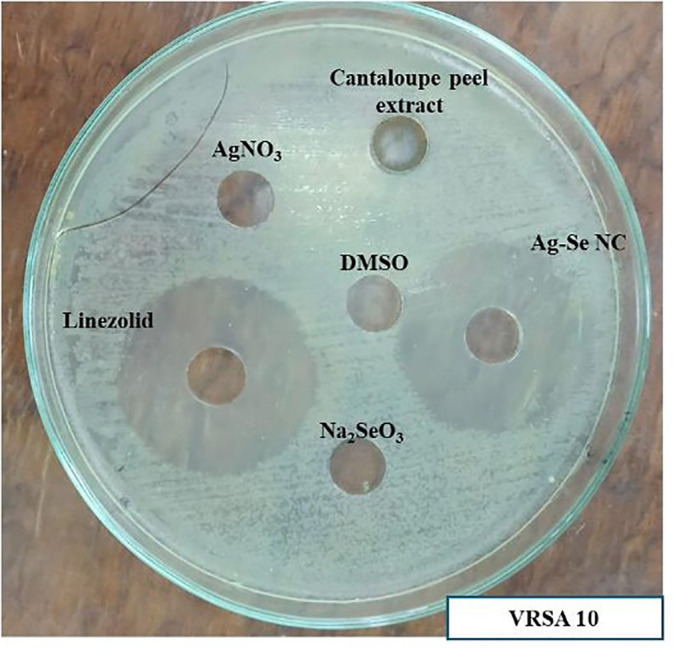
Antibacterial activity of the biosynthesized Ag–Se NC against the vancomycin-resistant Staphylococcus aureus (VRSA10) isolate as determined by the agar well diffusion (AWD) assay. The inhibitory effect of Ag–Se NC was compared with *Cucumis melo* L. peel extract, AgNO₃, Na₂SeO₃, linezolid as a positive control, and 10% DMSO as a negative control on Mueller–Hinton agar (MHA).

Based on the susceptibility of the tested *S. aureus* isolates to vancomycin, as determined by the broth microdilution method, 11/42 isolates (26.2%) were identified as VRSA and designated VRSA_1_-VRSA_11_. The AWD assay (Fig 7) illustrated the antibacterial activity of Ag–Se NC (1000 µg/mL) against the phenotypically identified VRSA isolates in comparison with the linezolid (30 µg/mL) positive control (Table 2). The IZD for the Ag–Se NC varied substantially across the isolates, from a minimum of 8.00 ± 0.00 mm (in case of VRSA_7_) to a maximum of 22.67 ± 1.15 mm (in case of VRSA_6_). Additionally, VRSA_11_ and VRSA_10_ demonstrated the highest relative sensitivities (90.5% and 83.1%, respectively) to the tested NC, while VRSA_7_ and VRSA_4_ were the least affected, 31.2% and 49.4%, respectively. In comparison, linezolid exhibited more consistent and generally larger zones, ranging from 23.00 ± 0.00 mm to 29.70 ± 0.60 mm. The PI of Ag–Se NC, calculated relative to the linezolid control for each isolate, highlighted a marked differential susceptibility. A statistically significant difference (*P* < 0.001) with greater linezolid potency towards VRSA isolates than Ag–Se NC was confirmed using a paired sample t-test. Also, Pearson’s correlation (r = 0.72, *P* = 0.012) indicated a positive relationship, suggesting shared susceptibility factors. The higher coefficient of variation for Ag–Se NC (27.5%) versus linezolid (7.8%) highlights marked isolate-dependent variability, emphasizing the need for tailored NP-based interventions.

**Table 2:** Inhibitory activity of Ag–Se NC against different VRSA isolates.

CharacterIsolates	Agar well diffusion assay		
	Ag–Se NC	Linezolid	PI (%)
	(1000 µg/mL)	(30 µg/mL)	
	IZD (mm)		
VRSA_1_	20.33 ± 0.58	25 ± 0	81.3
VRSA_2_	15 ± 0	25.3 ± 1.2	57.7
VRSA_3_	19.67 ± 0.58	25.3 ± 0.6	77.6
VRSA_4_	13.33 ± 0.58	27 ± 1	49.4
VRSA_5_	15 ± 0	27.3 ± 0.6	54.9
VRSA_6_	22.67 ± 1.15	29.7 ± 0.6	76.4
VRSA_7_	8 ± 0	25.7 ± 0.6	31.2
VRSA_8_	18.33 ± 0.58	23 ± 0	79.7
VRSA_9_	15 ± 0	27 ± 0	55.6
VRSA_10_	21.33 ± 0.58	25.7 ± 0.6	83.1
VRSA_11_	22.33 ± 0.58	24.7 ± 0.6	90.5

The observed variation in susceptibility among VRSA isolates is a common phenomenon, often attributed to differences in efflux pump activity and biofilm formation capabilities. These findings suggest a potentially enhanced NC mechanism of action beyond that of linezolid, which suppresses bacterial protein synthesis. A comparable study reported synergistic antibacterial effects of their biosynthesized bimetallic CuO-Se NPs towards MDR *Pseudomonas aeruginosa* (*P. aeruginosa*); the synergistic interaction may also explain the potent activity of our NC here [56]. The significant inhibitory zones, even at a concentration of 1000 µg/mL, are promising when compared to the high toxicity often associated with Ag NPs alone. Recent research highlighted that the incorporation of Se NPs mitigates the cytotoxic profile of Ag NPs while maintaining robust antimicrobial properties, a crucial advantage for potential therapeutic development [116]. Furthermore, a study on bimetallic Ag–Se NC concluded that the NC structure can lead to multifaceted mechanisms of action and hypothetically reduce the possible development of resistance in comparison with conventional antimicrobial agents [117]. These collective findings position Ag–Se NC as a compelling candidate for future development as an alternative or adjunctive therapeutic agent. It should be mentioned that the reference antimicrobial linezolid was utilized at 30 µg/mL, while Ag-Se BNPs was examined at 1000 µg/mL. As a result, the inhibition zone widths are intended to serve as qualitative preliminary markers of antibacterial activity rather than potency-equivalent comparisons. The data collectively underscore the potential of Ag–Se NC as a viable alternative or adjunct to conventional antimicrobials like linezolid, meriting further investigation into their specific mechanisms and *in vivo* efficacy.

### 3.11 Quantitative inhibitory concentrations

Prior to assessing the antibacterial activity of the produced nanocomposites, the disk diffusion assay was used to test the solvent (10% DMSO), *C. melo* L. peel extract, and the precursor salts, sodium selenite (1000 µg/mL) and silver nitrate (1000 µg/mL). The antibacterial results described here are solely traceable to the manufactured nanocomposites because none of these controls generated any discernible zone of inhibition against the tested microbial strains.

The inhibitory activity of biosynthesized Ag–Se NC against 11 VRSA clinical isolates was revealed by broth microdilution assays. The MIC ranged from 64 to 512 µg/mL, with a calculated MIC_50_ of 102.84 µg/mL. Isolates VRSA_6_, VRSA_10_, and VRSA_11_ exhibited the lowest MIC values (64 µg/mL each), indicating heightened susceptibility, while VRSA_7_ demonstrated the highest MIC (512 µg/mL), suggesting reduced sensitivity. Additionally, the MBCs varied between 128 and 1024 µg/mL and the MICi ranged from 1 to 4. Isolates VRSA_1_ and VRSA_7_ displayed MICi = 1, indicating strong bactericidal NC activity against these two isolates. Notably, isolates VRSA_3_, VRSA_5_, and VRSA_10_ also demonstrated strong bactericidal profiles despite MICi = 2, suggesting enhanced killing kinetics. The remaining isolates exhibited MICi = 4 indicating delayed bactericidal effects. These data highlight the differential susceptibility of VRSA strains to Ag–Se NC and underscore the potential of these nanomaterials as alternative antimicrobial agents (Table 3). Statistical analysis confirmed significant and strain-specific responses of VRSA isolates to Ag–Se NC (*P* < 0.001). One-way ANOVA for the MIC_50_ values across the tested isolates showed significant differences (*P* < 0.001), and Tukey’s post hoc test identified VRSA_6_ as significantly more resistant than VRSA_5_, VRSA_9_, VRSA_10_, and VRSA_11_ (*P* < 0.01).

**Table 3:** Inhibitory concentrations of Ag–Se NC against VRSA clinical isolates.

Test Isolates	Ag–Se NC broth microdilution assay				Antibiosis effect
	MIC	MIC_50_	MBC	MIC_i_	
	(µg/mL)				
VRSA_1_	256	102.84	256	1	Strong bactericidal
VRSA_2_	256	166.51	1024	4	Bactericidal
VRSA_3_	128	77.27	256	2	Strong bactericidal
VRSA_4_	256	156.77	1024	4	Bactericidal
VRSA_5_	256	118.89	512	2	Strong bactericidal
VRSA_6_	64	28.11	256	4	Bactericidal
VRSA_7_	512	320.82	512	1	Strong bactericidal
VRSA_8_	128	83.38	512	4	Bactericidal
VRSA_9_	256	129.19	1024	4	
VRSA_10_	64	43.2	128	2	Strong bactericidal
VRSA_11_	64	26.95	256	4	Bactericidal
Mean	203.64	113.99	523.64	–	–

In this study, it was observed that the inhibitory effects are a direct result of the nanocomposite formulation rather than any residual activity of its separate components, which comes from the lack of antimicrobial activity from the solvent, plant extract, and precursor salts. The antimicrobial performance of Ag–Se NC against VRSA isolates in this study was consistent with recent findings on NPs-based strategies targeting MDR pathogens. The fungal-mediated synthesized Ag NPs and Se NPs showed strong antibacterial and antibiofilm activities towards MRSA and MDR *P. aeruginosa*, with Se NPs showing stronger antibacterial effects and Ag NPs excelling in biofilm inhibition [118]. Also, our MIC_50_ value (102.84 µg/mL) falls within the range reported for similar NC, although slightly elevated compared to monometallic formulations, possibly due to the complex resistance mechanisms in VRSA strains [40]. This aligns with our observation of strong bactericidal effects in isolates VRSA_1_, VRSA_3_, VRSA_5_, VRSA_7_, and VRSA_10_, suggesting that Ag–Se NC may exert multifaceted antimicrobial actions, including membrane disruption and oxidative stress induction. The variation in MICi values among isolates further supports the hypothesis of strain-specific resistance determinants, such as efflux pump activity or biofilm architecture, influencing NPs susceptibility. Regarding the Ag NP mechanisms, a comprehensive review highlights their ability to interfere with bacterial metabolism, DNA replication, and protein synthesis [119]. Moreover, the observed MIC_50_ variability aligns with recent findings that reported MIC values between 25 and 40 µg/mL for Ag–Se NC against *S. aureus* and *E. coli*, with dose-dependent and significant biofilm inhibitory activity [120]. Similarly, those biosynthesized Ag_2_Se NPs demonstrated MICs ranging from 150–250 µg/mL against Gram-negative and Gram-positive strains, confirming the broad-spectrum efficacy of bimetallic formulations [121]. However, our findings diverge from the study that illustrated rapid bacterial inhibition at lower concentrations using tea-extract-enhanced Ag NPs, although their tested strains lacked the ability to resist vancomycin [122].

### 3.12 Bacterial biofilm inhibition and eradication activities

**Fig 8:**
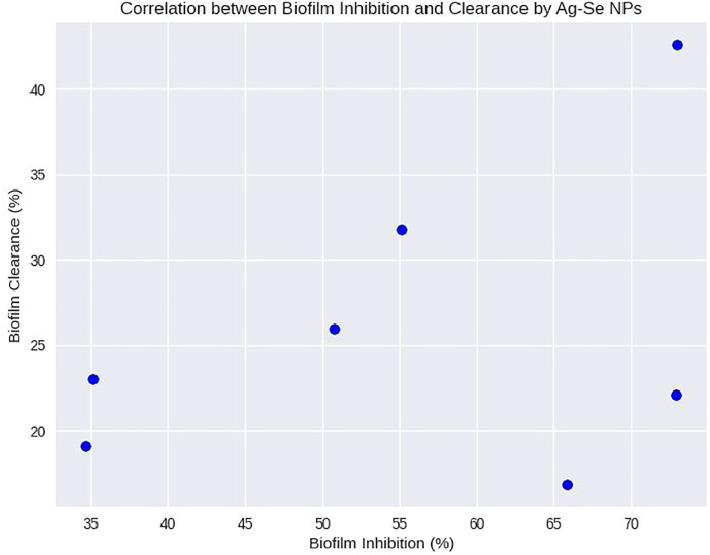
Relationship between the biofilm inhibition and biofilm clearance activities of the biosynthesized Ag–Se NC against vancomycin-resistant *Staphylococcus aureus* (VRSA) isolates, as determined by the crystal violet (CV) assay. Each data point represents an individual VRSA isolate, illustrating the association between inhibition and clearance percentages and the corresponding Pearson correlation analysis.

From our previous study [52], the tested VRSA isolates had belonged to different biofilm formation categories (Table 4). The biofilm inhibition and clearance activities of Ag–Se NC against 7 biofilm-forming VRSA isolates demonstrated variable inhibitory and eradication effects depending on the biofilm-forming capacity of the isolates. Isolates with weak biofilm formation (VRSA_1_ and VRSA_4_) exhibited relatively high inhibition (55.09% and 72.97%) and clearance (31.76% and 42.59%), and strong positive correlations (r = +0.62 and +0.84, respectively) were observed. Moderate biofilm formers (VRSA_3_ and VRSA_10_, respectively) showed inhibition values of 50.77% and 65.85%, but clearance was lower (25.94% and 16.81%), with correlations ranging from moderate positive (r = +0.58) to weak negative (r = –0.21). Strong biofilm formers (VRSA_2_, VRSA_5_, and VRSA_9_) displayed inconsistent responses: isolates VRSA_2_ and VRSA_9_, respectively, showed low inhibition (35.16% and 34.68%), clearance (23.02% and 19.11%), and strong positive correlations (r = +0.76 and +0.79), whereas isolate VRSA_5_ exhibited high inhibition (72.89%) but poor clearance (22.1%), resulting in a negative correlation (r = –0.15). The findings indicated that Ag–Se NC possess dual antibiofilm activity, both inhibiting biofilm formation and promoting clearance of established biofilms, although the strength of this effect varied across isolates. The positive correlations observed in most isolates suggest that inhibition and clearance are generally interrelated, yet exceptions such as VRSA_5_ and VRSA_10_ isolates highlight that strong inhibition does not always translate into effective eradication. Statistically, the one-way ANOVA confirmed significant differences among isolates (*P* < 0.05), underscoring the strain-dependent antibiofilm efficacy of Ag–Se NC. Additionally, Pearson correlation analysis revealed a weak, non-significant positive relationship between biofilm inhibition and clearance across VRSA isolates (r = 0.383, *P* = 0.396), suggesting limited predictive alignment. High variability in VRSA_5_ and VRSA_10_ isolates contributed to reduced correlation strength (Fig 8).

**Table 4:** Biofilm inhibition and clearance activities of Ag–Se NC against VRSA isolates.

Isolates	Cutoff Value	Biofilm formation	Ag–Se NC		
			Biofilm Inhibition (%)	Biofilm clearance (%)	Pearson’s *(r)*
VRSA_1_	1.057	Weak	55.09 ± 8.65	31.76 ± 4.34	+0.62
VRSA_2_	3.421	Strong	35.16 ± 2.3	23.02 ± 0.93	+0.76
VRSA_3_	2.552	Moderate	50.77 ± 11.24	25.94 ± 0.93	+0.58
VRSA_4_	1.333	Weak	72.97 ± 1.35	42.59 ± 0.78	+0.84
VRSA_5_	3.162	Strong	72.89 ± 1.87	22.1 ± 0.69	–0.15
VRSA_6_	0.381	None	NA	NA	NA
VRSA_7_	0.624	None	NA	NA	NA
VRSA_8_	0.512	None	NA	NA	NA
VRSA_9_	3.428	Strong	34.68 ± 2.4	19.11 ± 0.76	+0.79
VRSA_10_	2.815	Moderate	65.85 ± 0.67	16.81 ± 0.96	–0.21
VRSA_11_	0.355	None	NA	NA	NA

NA: Not applicable

Our findings agreed with previous studies on the antibiofilm potential of metallic NPs. For instance, the biosynthesized Fe-Ag BNCs suppress *S. aureus* and *P. aeruginosa* biofilms, with efficacy linked to NPs composition and ion release dynamics [123]. Similarly, recent studies on Ag–Se NC synthesized via microbial or gamma irradiation methods have confirmed their strong antibiofilm activity against Gram-positive and Gram-negative pathogens, with inhibition rates often exceeding 60–70% [124]. In the same context, the present results further corroborate the work demonstrating Ag–Se NC effective eradication of MRSA biofilms, a key virulence factor in bacterial infections [125]. The present findings align with these observations, reinforcing the potential of Ag–Se NC as promising antibiofilm agents, particularly against MDR *staphylococci*.

### 3.13 Time of kill assay of Ag–Se NC against VRSA_10_ isolate

**Fig 9:**
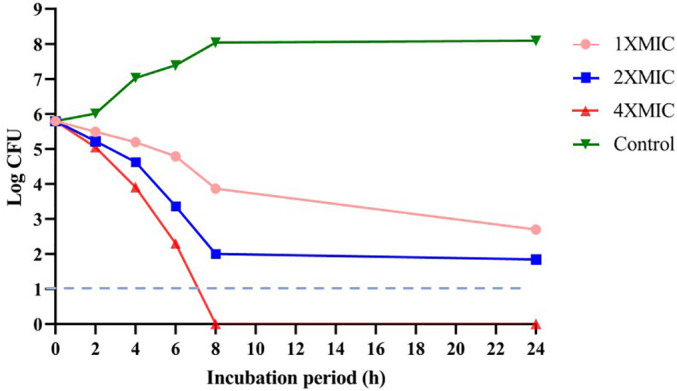
Time–kill kinetics of the biosynthesized Ag–Se NC against the VRSA10 isolate at MIC, 2 × MIC, and 4 × MIC over a 24-h incubation period. Bacterial survival was monitored by viable cell counting, and the dashed line represents the limit of detection (LOD) of the plate count method. The bactericidal activity of Ag–Se NC was evaluated based on reductions in viable bacterial counts relative to the initial inoculum.

The bacterial inhibitory activity of Ag–Se NC at MIC, 2 × MIC, and 4 × MIC was obvious towards the VRSA10 clinical isolate that showed the highest antimicrobial activity (IZD = 21.33 ± 0.58, MIC = 64, MIC_50_ = 43.2, and MICi = 2) using a time of kill assay. The initial bacterial load at 0 hrs was 5.792 log CFU/mL across all groups. The viable counts were reduced in a concentration-dependent manner over time. At MIC, log CFU decreased gradually to 2.699 by 24 hrs, indicating moderate bacteriostatic activity. At 2 × MIC, a more pronounced decline was recorded, reaching 1.845 log CFU/mL at 24 hrs. Notably, complete bacterial eradication was achieved at 4 × MIC by 8 hrs, with no detectable CFU through 24 hrs. In contrast, the untreated control group showed progressive bacterial growth, increasing from 5.792 to 8.093 log CFU/mL over 24 hrs. These findings confirm the potent and rapid bactericidal effect of Ag–Se NC against VRSA_10_, particularly at higher concentrations (Fig 9). Statistical analysis demonstrated significant main effects for both NC concentration and exposure time (*P* < 0.001), as well as a strong interaction between the two factors (*P* < 0.001), indicating that bacterial killing by Ag–Se NC depends on both dose and duration.

Notably, the successfully biosynthesized Ag–Se NC via a green approach utilizing endophytic fungi demonstrated their potent activity against *S. aureus* [120]. The NC exhibited a MIC of 30 µg/mL, alongside a 70% inhibition of biofilm formation. A similar study reviewed metallic NPs and emphasized that size, surface charge, and synthesis method critically influence antibacterial efficacy. They noted that Ag NPs and Au NPs are effective against Gram-positive bacteria, including resistant strains [126].

### 3.14 Protein leakage assay

**Fig 10:**
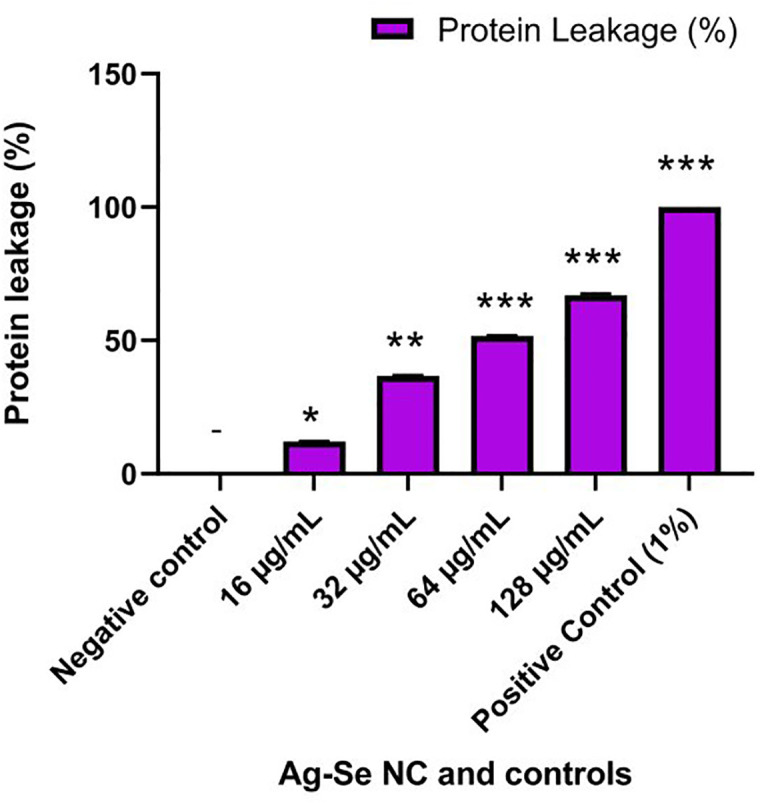
Effect of the biosynthesized Ag–Se NC on protein leakage from VRSA10 cells following treatment with different concentrations of the nanocomposite. Extracellular protein levels were quantified using the Bradford assay, and the results are presented as percentages of protein leakage relative to the appropriate controls. Statistical significance is indicated by asterisks.

The protein leakage assay demonstrated a concentration-dependent increase in membrane disruption of VRSA_10_ following treatment with Ag–Se NC (Fig 10). The negative control (untreated cells) exhibited a baseline protein concentration of 0.54 ± 0.034 µg/mL. Treatment with Ag–Se NC at 1/4x MIC (16 µg/mL) induced a measurable leakage, with protein levels rising to 10.08 ± 0.059 µg/mL, corresponding to 12.03% of the maximum leakage observed with Triton X-100 (1% v/v). This effect intensified considerably at higher concentrations. Exposure to 1/2x MIC (32 µg/mL) and the full MIC (64 µg/mL) resulted in protein concentrations of 29.66 ± 0.094 µg/mL (36.71%) and 41.49 ± 0.094 µg/mL (51.63%), respectively. The most severe membrane damage occurred at 2x MIC (128 µg/mL), where the leaked protein concentration reached 53.65 ± 0.49 µg/mL, representing 66.97% of the total lysis achieved by the positive control (79.85 ± 0.78 µg/mL). Statistically, our observations showed a clear positive correlation between Ag–Se NC concentration and protein leakage (*r* = 0.7221), in addition we used the one-way ANOVA (to compare all treatment groups against each other) followed by post-hoc Tukey’s test to specifically compare each concentration against the negative control. The narrow standard deviations across all samples indicate high reproducibility and precision in the experimental measurements. The significant leak in protein leakage between the 1/2x MIC and MIC concentrations, alongside the continued increase at 2x MIC, confirms a statistically robust and concentration-dependent effect rather than a random or saturating trend.

The observed concentration-dependent escalation in protein leakage provides unequivocal evidence that the primary bactericidal mechanism of Ag–Se NC against VRSA may be the disruption of cytoplasmic membrane integrity. This conclusion is strongly supported by prior research on metal and metalloid NPs. For instance, Ag NPs induce membrane permeability in MRSA, leading to ATP efflux and cell death, a finding directly analogous to the protein leakage demonstrated here [127]. As demonstrated by our protein leakage assay, which showed up to 66.97% leakage at 2 × MIC, the results of the current study indicate that biogenic Ag-Se NC exerts its antibacterial activities against VRSA through many synergistic routes, including ROS production and membrane disruption. By generating hydroxyl radicals and superoxide anions, which harm bacterial DNA, proteins, and lipids, silver ions from Ag-Se NCs cause oxidative stress. Selenium components exacerbate this by catalase suppression and further ROS imbalance. In line with our time-kill kinetics showing quick bactericidal activity (≥3 log reduction at 4 × MIC within 8 hours), membrane permeabilization happens as NPs stick to lipid bilayers, creating pits and allowing intracellular contents like proteins and ATP to seep out [128,129]. Accordingly, the efficacy of Ag–Se NC against the tested VRSA is of critical importance, as this mechanism circumvents the traditional antimicrobial resistance pathways, offering a promising physical strategy to combat MDR strains.

### 3.15 Synergistic antibacterial activity

**Fig 11:**
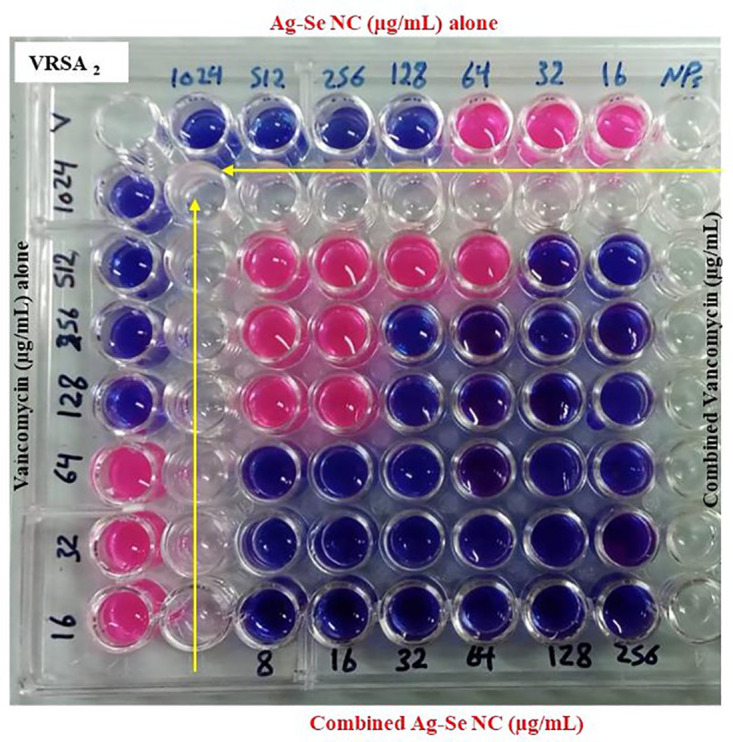
Heatmap of the fractional inhibitory concentration index (FICi) generated from a resazurin-based checkerboard assay evaluating the interaction between vancomycin and the biosynthesized Ag–Se NC against the VRSA2 isolate. The heatmap presents the antimicrobial response across different concentration combinations of both agents.

The checkerboard assay results presented for 11 tested VRSA isolates provide a nuanced evaluation of the interaction between vancomycin and Ag–Se NC (Fig 11). Based on calculated FIC, the combination yielded varied outcomes: the tested VRSA_2_ isolate exhibited clear synergism with FICi = 0.5, three isolates showed additive effects based on the FICi value between (0.5–1), and the remaining seven isolates demonstrated indifference effects with FICi > 1 (Table 5). Notably, isolate VRSA_2_ displayed the most pronounced synergy, with both agents reducing their MICs by 2-fold when combined. This heterogeneity underscores the strain-dependent nature of NC-antimicrobial interactions and highlights the therapeutic potential of Ag–Se NC as adjuncts to vancomycin, particularly in selected VRSA phenotypes.

**Table 5:** Combined effect of Ag–Se NC and vancomycin against VRSA isolates.

Isolates	Combined MIC		Separated MIC		FIC		FICi	Combined effect
	Ag–Se NC	Vancomycin	Ag–Se NC	Vancomycin	Ag–Se NC	Vancomycin		
VRSA_1_	128	128	256	128	0.5	1	1.5	Indifference
VRSA_2_	64	32	128	128	0.25	0.25	0.5	Synergism
VRSA_3_	64	128	128	512	0.5	0.125	0.625	Additive
VRSA_4_	256	64	256	256	1	0.25	1.25	Indifference
VRSA_5_	256	128	256	256	1	0.125	1.125	
VRSA_6_	64	128	64	256	1	0.125	1.125	
VRSA_7_	256	128	512	256	0.5	0.125	0.625	Additive
VRSA_8_	64	256	128	128	0.5	2	2.5	Indifference
VRSA_9_	64	256	256	256	0.25	0.5	0.75	Additive
VRSA_10_	32	128	64	128	2	0.031	2.031	Indifference
VRSA_11_	64	256	64	128	1	0.25	1.25	

The observed synergy in the VRSA_2_ isolate aligns with a related study’s findings, which reported a strong synergistic effect (FIC = 0.37) between biogenic Ag NPs and vancomycin towards MRSA, both *in vitro* and *in vivo* [130]. Numerous studies have investigated the NPs-antimicrobial agent combinations against MDR microbes. For instance, Ag–Se NC combined with linezolid demonstrated reduced MICs against MRSA by 4-fold, though the FICi hovered around 0.75, indicating partial synergy [131,132]. Similarly, Se NPs enhanced the bactericidal activity of β-lactams against Gram-positive cocci, but without achieving full synergism, the FICi ~ 0.6 [118]. In contrast, the current findings present a more robust synergistic profile (FICi = 0.5), aligning with the research recorded complete synergy between Ag NPs and vancomycin against VRSA isolates, albeit with higher MIC values [40]. The integration of Se into the bimetallic matrix may confer enhanced redox activity and membrane disruption potential, thereby amplifying vancomycin uptake. This supports the hypothesis that Ag–Se NC offers a superior platform for combinatorial antimicrobial therapy, particularly against vancomycin-resistant phenotypes.

**Fig 12:**
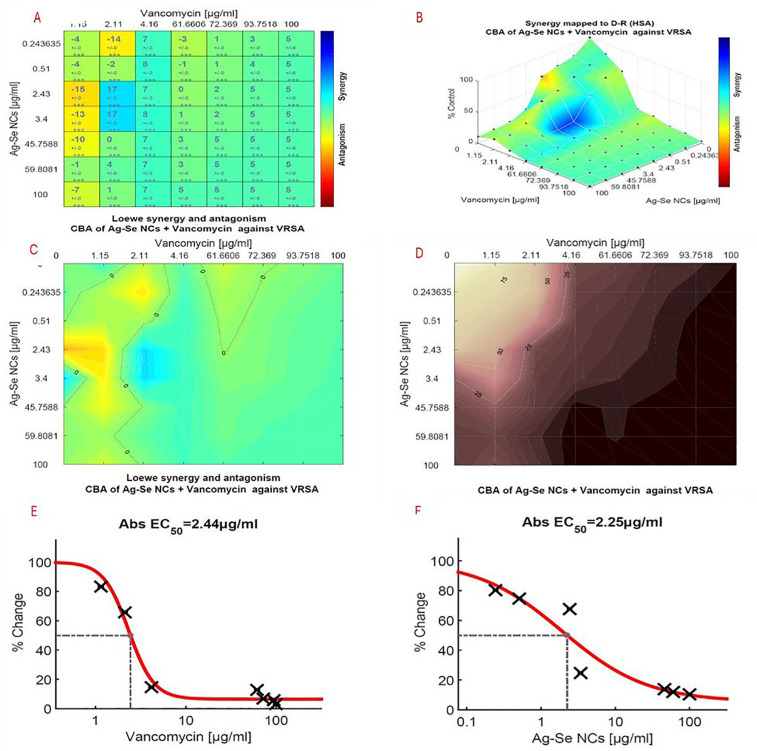
Analysis of the interaction between vancomycin and the biosynthesized Ag–Se NC against the VRSA2 isolate using Combenefit® software. Panels A–D present synergy and antagonism maps generated using different interaction models, illustrating the combined effects of both agents across a range of concentrations. Panels E and F show the dose–response curves of vancomycin and Ag–Se NC, respectively, used for the assessment of drug interaction and potency.

Additionally, the checkerboard assay results for the VRSA_2_ isolate, analysed using Combenifit^®^ software, demonstrate a pronounced synergistic interaction between vancomycin and Ag–Se NC (Fig 12). The combination yielded a FICi of 0.5, confirming synergism according to the accepted interpretive criteria. Based on the Combenefit’s multi-model analysis, panels A through D illustrate synergy landscapes generated from distinct analytical models, each mapping concentration-dependent interactions across a matrix of vancomycin and Ag–Se NC doses. These models consistently reveal zones of positive synergy, particularly within intermediate concentration ranges, suggesting enhanced antibacterial efficacy when both agents are co-administered. Panels E and F display dose-response curves for vancomycin and Ag–Se NC, respectively, with calculated EC_50_ values of 2.44 µg/mL and 2.25 µg/mL, indicating enhanced potency when used in tandem. The integration of these analyses supports the conclusion that Ag–Se NC potentiates vancomycin activity against resistant strains, offering a promising combinatorial approach for mitigating therapeutic failure in VRSA infections. Recently, the mechanistic basis of Ag NPs was reviewed, illustrating the capability for disrupting bacterial membranes and biofilms, facilitating antibiotic penetration, and reducing MIC thresholds [40]. While these studies focused on monometallic Ag systems, the incorporation of Se in the current bimetallic formulation may confer additional oxidative stress and membrane destabilization, amplifying vancomycin uptake [133]. Further emphasized the utility of combinatorial approaches, showing that antimicrobials paired with vancomycin yielded enhanced synergy against resistant bacteria, particularly when evaluated via checkerboard and surface interaction models.

## 4 Conclusion

This study demonstrates the successful green synthesis of Ag–Se NC using *C. melo* L. peel extract as a sustainable reducing and stabilizing agent, emphasizing the value of agricultural waste in nanotechnology applications. The biosynthesized Ag–Se NC showed well-defined physicochemical characters, prominent crystallinity, nanoscale dimensions, and high dispersion stability, confirming the effectiveness of the biogenic synthesis route. Biological investigations revealed that Ag–Se NC possess strong anticancer effects against MCF-7 and Hep-G2 cancer cell lines. Additionally, the nanocomposites displayed potent antibacterial activity against VRSA clinical isolates, including rapid bactericidal action, membrane damage, and protein leakage, indicating a mechanism that bypasses conventional resistance pathways. Their ability to inhibit biofilm formation and partially eradicate established biofilms further underscores their therapeutic relevance, particularly in persistent and device-associated infections. Therefore, these results position Ag–Se NC as a multifunctional nanomaterial with significant promise for combating MDR bacterial infections and cancer. Future in vivo studies and mechanistic investigations are warranted to further validate their safety, efficacy, and clinical applicability.

## 5 Limitations of the study and future work

Despite the encouraging results, it is important to recognize the study’s limitations. First, all biological analyses were carried out in vitro, which might not accurately reflect the intricate host-pathogen relationships and pharmacokinetic characteristics. Second, only one of the eleven tested isolates showed the synergistic interaction with vancomycin, indicating that the antibiotic-potentiating effect may not be generally applicable. Third, in vivo toxicological profiling including pharmacokinetics, biodistribution, organ-specific toxicity, hemocompatibility, and long-term safety assessments was not carried out. Fourth, additional molecular-level analysis, such as transcriptome or proteomic profiling, would offer a deeper understanding of the specific bactericidal pathway. Fifth, future research will include ROS quantification, apoptosis assays, oxidative stress markers, mitochondrial membrane potential analysis, and gene/protein expression profiling to experimentally validate the mechanistic pathways suggested for the antibacterial and anticancer activities of the Ag–Se nanocomposite, such as ROS generation, oxidative stress, membrane disruption, and apoptosis induction. Lastly, the work used a single plant source (C. melo L. peel extract) for green synthesis, and biological performance and nanocomposite reproducibility may be impacted by batch-to-batch variations in phytochemical composition.

Subsequent research will concentrate on clarifying the fundamental mechanism of action, evaluating cytotoxicity, and appraising the effectiveness of the nanocomposites against a wider range of vulnerable and resistant infections. In addition, a comparative evaluation of nanocomposite efficacy against vancomycin-susceptible S. aureus as well as other clinically relevant pathogens is planned as part of our future investigations. Also, future research will also examine the following: (i) the cytotoxicity of the Ag–Se NC–vancomycin combination on normal cell lines at the effective synergistic concentrations; (ii) the combined treatment’s haemolysis and hemocompatibility; and [134] in-vivo efficacy and safety studies using suitable animal infection models to confirm the translational potential of this combinatorial approach. Additionally, incorporation of SEM/TEM analysis of nanocomposite-treated VRSA cells in future studies to provide direct morphological evidence of the membrane-disrupting mechanism. Moreover, conducting the time-kill analysis on isolates representing different susceptibility categories to capture the full spectrum of bactericidal dynamics.
